# Significance-Aware Federated Reinforcement Learning for AoI Optimization of Vehicular Sensing in UAV-Assisted Edge Networks

**DOI:** 10.3390/s26185783

**Published:** 2026-09-11

**Authors:** Xueyuan Wang, Siyu Bai, Yu Zhang, Mustafa C. Gursoy

**Affiliations:** 1 School of Computer Science and Artificial Intelligence, Changzhou University, Changzhou 213164, China; s24150812001@smail.cczu.edu.cn (S.B.); s23150812064@smail.cczu.edu.cn (Y.Z.); 2 Department of Electrical Engineering and Computer Science, Syracuse University, Syracuse, NY 13244, USA; mcgursoy@syr.edu

**Keywords:** vehicular sensing, age of information, UAV-assisted edge networks, federated learning, multi-agent reinforcement learning

## Abstract

Timely vehicular sensing is important for traffic monitoring, cooperative driving, and road-safety management. High mobility, time-varying wireless conditions, and limited edge resources nevertheless make information freshness difficult to maintain. This paper studies age of information (AoI) minimization in a three-layer UAV-assisted edge network comprising vehicle devices (VDs), unmanned aerial vehicles (UAVs), and a cloud center (CC). VDs periodically generate sensor-data packets, UAVs provide mobile edge processing and data-relaying services, and the CC coordinates system-wide resource allocation. The joint optimization of sensor-data transmission, UAV movement, packet processing, computation offloading, and bandwidth allocation is formulated within a cooperative multi-agent framework. To solve this problem, we propose a collaborative heterogeneous federated actor–critic (CHFAC) framework. Its significance-aware federated learning mechanism evaluates local model updates according to update significance, alignment with the global learning direction, and training stability and uses the resulting contribution scores for non-uniform agent selection and contribution-weighted aggregation. In the considered simulation setting, evaluation over 1000 test episodes yields an average AoI of 7.45±1.65 and a worst-case AoI of 38.72±24.06. The average AoI is 79.0%, 63.9%, and 29.2% lower than that obtained by the implemented HF-MARL, H-MAAC, and non-federated baselines, respectively. These results demonstrate the effectiveness of CHFAC for freshness-aware vehicular sensing in dynamic UAV-assisted edge environments.

## 1. Introduction

The rapid development of the Internet of Things (IoT) has enabled extensive sensing and information-processing applications in smart cities, industrial automation, and intelligent transportation systems [1]. As an important IoT application, the Internet of Vehicles (IoV) integrates vehicles, roadside infrastructure, communication networks, and computing platforms into a cooperative vehicular sensing environment [2]. Modern vehicles equipped with onboard sensors continuously generate time-sensitive data related to their positions, velocities, operating states, and surrounding traffic conditions, supporting applications such as cooperative driving, traffic monitoring, collision avoidance, and road-safety management [3]. Although Vehicle-to-Everything (V2X) communication enables the exchange of sensing information among vehicles, infrastructure, and remote control centers [4], high vehicle mobility, time-varying wireless channels, and limited onboard computing capabilities make timely data transmission and processing challenging. Mobile edge computing (MEC) alleviates this problem by deploying computing resources closer to vehicle devices (VDs) [5]. However, fixed terrestrial edge infrastructure may provide insufficient coverage or computing capacity in dynamic traffic environments. Owing to their flexible deployment, controllable mobility, and wide communication coverage, unmanned aerial vehicles (UAVs) can serve as mobile edge nodes to collect, process, and relay vehicular sensor-data packets to a cloud center (CC) [6,7].

For real-time vehicular sensing, conventional transmission delay cannot fully characterize the timeliness of the information available at the CC. The age of information (AoI), which measures the elapsed time since the generation of the most recently received status update, provides an appropriate metric for evaluating sensing-information freshness [8,9]. Maintaining a low AoI is particularly important for safety-critical applications such as autonomous driving, which rely on up-to-date information about vehicle positions and velocities [10]. Minimizing AoI in UAV-assisted vehicular sensing networks requires the joint optimization of sensor-data transmission, UAV movement, edge packet processing, computation offloading, and bandwidth allocation. This problem is challenging because of heterogeneous system components, high-dimensional decision spaces, vehicle mobility, and time-varying communication conditions. Multi-agent reinforcement learning (MARL) enables distributed agents to learn cooperative policies through interaction with the dynamic environment [11]. Nevertheless, independently trained agents have limited knowledge-sharing capabilities, whereas centralized learning requires the collection of large volumes of local vehicular data. Federated learning (FL) enables distributed agents to collaboratively train learning models while retaining their raw observations and experiences locally [12,13]. However, conventional federated approaches commonly employ random participant selection and uniform model aggregation without considering the learning value and stability of individual local updates, which may slow convergence in heterogeneous vehicular sensing environments.

To address these limitations, this paper proposes a significance-aware federated reinforcement learning framework for AoI optimization of vehicular sensing in UAV-assisted edge networks. The main contributions are summarized as follows:

- We develop a three-layer UAV-assisted edge architecture for timely vehicular sensing. The average AoI minimization problem is formulated by jointly optimizing local computation, UAV movement, edge packet processing, computation offloading, and bandwidth allocation within a cooperative multi-agent framework.
- We propose a collaborative heterogeneous federated actor–critic (CHFAC) framework to address the resulting mixed discrete–continuous action space. VDs, UAVs, and the CC act as heterogeneous agents and cooperatively learn decision policies for improving the freshness and processing efficiency of vehicular sensor data.
- We design a significance-aware FL mechanism that evaluates local model updates according to update significance, alignment with the global learning direction, and training stability. The resulting contribution scores guide non-uniform agent selection and contribution-weighted parameter aggregation, thereby improving collaborative learning efficiency in heterogeneous sensing environments.
- We conduct comprehensive simulations to evaluate the proposed framework. Over 1000 test episodes, CHFAC achieves an average AoI of 7.45±1.65 and a worst-case AoI of 38.72±24.06. Compared with HF-MARL, H-MAAC, and the non-federated ablation, CHFAC reduces the average AoI by 79.0%, 63.9%, and 29.2%, respectively.

## 2. Related Work

This section reviews existing studies related to the proposed framework from four perspectives: UAV-assisted edge computing for vehicular sensing, AoI optimization in vehicular sensing networks, multi-agent reinforcement learning for dynamic edge networks, and federated reinforcement learning for distributed edge intelligence.

### 2.1. UAV-Assisted Edge Computing for Vehicular Sensing

The integration of UAVs into MEC systems has been extensively studied to provide flexible, on-demand, and reliable communication and computation services, especially in areas with limited terrestrial infrastructure. Early works focused on optimizing fundamental system trade-offs. For instance, Ndikumana et al. [14] developed a comprehensive framework to jointly manage communication, computation, caching, and control. More recently, Hao et al. [6] jointly optimized multi-UAV trajectories, binary task-offloading decisions, and communication and computation resources and developed a deep reinforcement learning solution for the resulting hybrid action space. Building on this, several studies have delved deeper into specific optimization aspects. Liu et al. [7] proposed a cooperative optimization framework to improve energy efficiency by jointly tuning UAV flight paths and resource allocation. Security has also become a major concern; Xu et al. [15] introduced a framework that integrates resource optimization with trajectory design to defend against security threats. To handle the uncertainty and stochastic nature of wireless environments, Yang et al. [16] developed an online algorithm for trajectory and resource optimization in stochastic UAV-enabled MEC systems.

Furthermore, some research has explored multi-UAV cooperation to enhance system capabilities. For example, Zhan et al. [17] jointly optimized UAV trajectories, service indicators, resource allocation, and computation offloading in a multi-UAV-enabled MEC system for time-constrained IoT applications. Another work by Xia et al. [18] jointly optimized UAV association, task splitting, and UAV trajectories in a cellular-connected multi-UAV MEC system to minimize the total energy consumption of the UAVs. Although these studies have established an important foundation for UAV-assisted edge computing, their primary objectives are generally throughput maximization, energy minimization, or latency reduction. The freshness of timestamped vehicular sensing updates, as characterized by AoI, has received comparatively less attention.

Recent studies have further investigated UAV motion and autonomous path planning under practical dynamic and geometric constraints. Lv et al. [19] developed a finite-time control scheme for a coaxial tilt-rotor UAV to improve flight stability under nonlinear dynamics and disturbances. Zhou et al. [20] proposed a quality-guaranteed Dubins path-planning method that explicitly considers obstacle avoidance and curvature constraints. Their subsequent review [21] systematically summarized the development of curvature-constrained Dubins planning, including obstacle-aware and real-time extensions. These studies mainly focus on flight-level control and geometric trajectory feasibility, whereas the present work considers UAV mobility as a network-level decision jointly coordinated with vehicular sensing, edge processing, computation offloading, and bandwidth allocation for AoI optimization.

### 2.2. AoI Optimization in Vehicular Sensing Networks

To quantify data freshness, the AoI has emerged as a critical performance metric [22], measuring the time elapsed since the generation of the most recently received information packet. Minimizing AoI is crucial for IoV and other real-time systems. Initial research focused on theoretical analysis of AoI in queuing systems [9]. More recently, research has shifted towards practical optimization in complex networks. Bai et al. [10] applied deep reinforcement learning (DRL) to manage AoI in vehicular networks by optimizing scheduling and power allocation. Goudarzi et al. [23] investigated AoI-aware UAV-assisted vehicular edge computing and employed a soft actor–critic method to jointly optimize UAV trajectories, vehicle association, and computation offloading. Han et al. [24] analyzed the impact of AoI on data collection in IoT systems. Qin et al. [25] examined AoI-aware scheduling in air–ground collaborative networks to ensure timely updates across diverse users.

The concept of AoI has also been extended to different contexts. Chi et al. [22] introduced the “Age of Model”, a metric for evaluating the freshness of AI models in edge intelligence-enabled IoV applications. Some works have also considered the trade-off between AoI and other metrics, such as energy consumption. Pei et al. [26] considered multiple transmission modes in UAV-assisted edge computing and minimized the average AoI subject to sensor energy constraints through joint communication and computational resource allocation. However, many of these works either rely on simplified system models or use traditional optimization methods that struggle with the high-dimensional, non-convex, and dynamic nature of our problem, which involves the joint optimization of UAV mobility, multi-user resource allocation, and computation scheduling. This complexity motivates the use of more advanced, adaptive techniques like MARL.

### 2.3. Multi-Agent Reinforcement Learning for Dynamic Edge Networks

MARL has demonstrated considerable potential for solving complex decision-making problems in dynamic multi-agent systems [11]. By interacting with the environment, distributed agents can learn cooperative or competitive policies without relying on complete prior knowledge of system dynamics. In communication and edge networks, MARL has been widely applied to resource allocation [27], interference management [28], computation offloading, and other distributed control problems. These capabilities make MARL a promising approach for coordinating heterogeneous agents in highly dynamic vehicular edge environments.

However, applying MARL to large-scale IoV systems remains challenging. First, fully centralized training frameworks, in which a single controller determines the actions of all agents, suffer from poor scalability, a single point of failure, and an excessively large joint state–action space, as discussed by Liu et al. [29]. Second, both centralized and decentralized MARL approaches may require agents to exchange local observations, trajectories, or experiences for coordinated training. Such information exchange may expose sensitive local data and incur considerable data-transfer costs in large-scale vehicular environments. These limitations motivate the development of distributed collaborative learning mechanisms that can facilitate knowledge sharing among agents while reducing the dependence on direct exchange of local observations and experiences.

Learning-based methods have also been extended to heterogeneous multi-agent control and autonomous decision-making. Valadbeigi et al. [30] developed a model-free Q-learning framework for robust output consensus in heterogeneous multi-agent systems without requiring explicit knowledge of the system dynamics. Wang et al. [31] employed an improved soft actor–critic framework for real-time autonomous obstacle avoidance under dynamic environmental constraints. These studies demonstrate the capability of reinforcement learning to handle uncertain and dynamic decision processes. In contrast, CHFAC addresses cooperative network-level optimization, where heterogeneous agents jointly manage UAV mobility, sensing-data processing, offloading, and communication resources while minimizing AoI.

### 2.4. Federated Reinforcement Learning for Distributed Edge Intelligence

To mitigate these privacy and scalability concerns, FL has been introduced as a distributed machine learning paradigm [32]. FL allows multiple clients to collaboratively train a shared model while keeping their raw data localized, only sharing model updates (e.g., gradients). This approach has been widely explored for various applications in wireless networks [13].

The integration of FL with MARL, commonly referred to as federated reinforcement learning (FRL), provides a distributed approach to collaborative policy training. For example, Zhu et al. [33] proposed H-MAAC, a federated multi-agent actor–critic framework for age-sensitive MEC. Li et al. [34] proposed a heterogeneous federated multi-agent reinforcement learning (HF-MARL) algorithm for AoI minimization in a three-layer multi-UAV-assisted MEC system. Their method jointly optimizes stochastic computation offloading, UAV trajectories, and communication-bandwidth allocation through heterogeneous actor–critic agents and periodic type-wise federated parameter updating. However, HF-MARL employs a preset parameter-mixing rule among ground-device and UAV agents of the same type and does not incorporate significance-aware participant selection or contribution-weighted aggregation. Therefore, HF-MARL is adopted as a representative external benchmark for evaluating the proposed CHFAC framework. Wu et al. [35] proposed a federated reinforcement learning framework for large-model task offloading and resource allocation in vehicular edge computing (VEC). Existing FRL frameworks often adopt standard protocols such as FedAvg, with uniform random client selection and uniform aggregation. Such protocols do not distinguish updates by directional consistency or temporal stability, and they may be sensitive to statistical heterogeneity (non-IID data) across agents [36]. In heterogeneous environments, less informative or unstable updates can therefore receive the same weight as well-aligned updates.

Model-based distributed optimization provides another paradigm for coordinating multiple agents under uncertainty. Li et al. [37] developed a distributionally robust self-triggered model predictive control framework for stochastic systems, while Li et al. [38] further investigated distributed, distributionally robust model predictive control under uncertain disturbances. Such methods provide structured optimization and robustness analysis when suitable system models are available. In comparison, federated reinforcement learning is more suited to the considered time-varying vehicular environment because policies can be learned directly through interaction without repeatedly solving an explicit online optimization problem. Moreover, CHFAC keeps local observations at individual agents and exchanges model updates for collaborative learning. Its significance-aware mechanism further distinguishes local updates according to their informativeness, directional consistency, and stability before federated aggregation.

Overall, existing studies have separately advanced UAV-assisted edge computing, AoI-aware resource optimization, MARL, and federated reinforcement learning. However, efficient federated collaboration among heterogeneous network agents remains insufficiently explored for freshness-sensitive vehicular sensing. In particular, conventional federated MARL generally does not explicitly distinguish local updates according to their learning significance, directional consistency, and training stability. Consequently, less informative or unstable updates may participate in global aggregation with comparable influence. This limitation motivates the proposed CHFAC framework, which incorporates significance-aware participant selection and weighted aggregation into heterogeneous actor–critic learning.

Table 1 provides a systematic comparison of the studies reviewed in this section in terms of UAV-assisted edge computing, information freshness, joint optimization, reinforcement learning, federated learning, and significance-aware updating.

## 3. System Model and Problem Formulation

**Figure 1:**
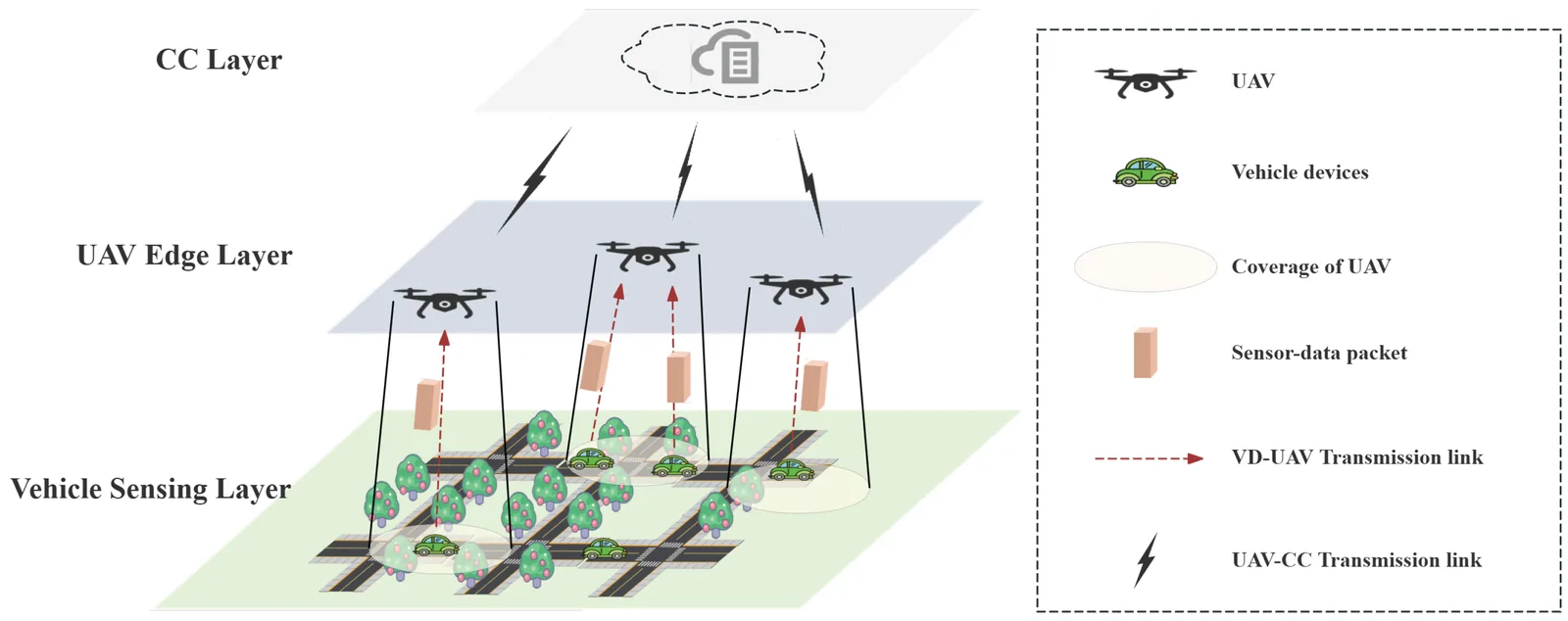
Three-layer UAV-assisted edge architecture for vehicular sensor-data collection, edge processing, and cloud-based information updating.

This section presents the considered three-layer UAV-assisted edge network for vehicular sensing, as illustrated in Figure 1. The first tier is the vehicle sensing layer, which consists of multiple VDs equipped with onboard sensing and processing capabilities. These VDs periodically generate sensor-data packets containing information about their operating states and surrounding traffic environments. The second tier is the UAV edge layer, in which multiple UAVs collect, process, and relay the sensor-data packets generated by the VDs. The upper tier is the CC, which coordinates system-wide bandwidth allocation and receives the processed sensor data. Time is divided into discrete slots indexed by *t*, and the main notation is summarized in Table 2.

### 3.1. Vehicular Sensor-Data Generation Model

In the considered vehicular sensing network, each VD is equipped with onboard sensing and processing units. These units periodically generate data related to the vehicle’s position, velocity, operating status, and surrounding traffic environment. The generated sensor data are encapsulated into packets for subsequent processing and transmission.

Each sensor-data packet generated by VD *m* at time slot *t* is represented as a tuple consisting of the packet size ($L_{m}\left(t\right)$), the time elapsed since its generation ($w_{m}\left(t\right)$), and a unique source identifier ($id_{m}$). The source identifier enables the system to distinguish and track packets generated by different VDs. Before computation or offloading, the generated packets are temporarily stored in the local source buffer.

### 3.2. Mobility Model

In the considered UAV-assisted vehicular sensing network, a set of *K* UAVs provides mobile edge coverage for spatially distributed VDs. Operating at a specified altitude (*H*) above the sensing sources, the position of the *k*-th UAV at time slot *t* is represented by $\left(X_{k}\left(t\right),Y_{k}\left(t\right),H\right)$. The movement of the *k*-th UAV during the *t*-th time slot can be modeled as follows:

(1) $$p_{k}\left(t+1\right)=p_{k}\left(t\right)+{\mathrm{move}}_{k}\left(t\right),$$

subject to the following constraint:

(2) $${∥{\mathrm{move}}_{k}\left(t\right)∥}_{2}\leq r_{\mathrm{move}}^{k},$$

where ${∥\cdot ∥}_{2}$ represents the Euclidean norm of the movement vector. Additionally, each VD, indexed by *m*, can proceed, reverse, and maneuver at intersections according to traffic and navigation needs, maintaining a consistent speed throughout its journey. The position of the *m*-th VD at time *t* is denoted as $\left(X_{m}\left(t\right),Y_{m}\left(t\right),0\right)$, with its movement dynamics described as follows:

(3) $$p_{m}\left(t+1\right)=p_{m}\left(t\right)+{\mathrm{move}}_{m}\left(t\right),$$

where $p_{m}\left(t\right)$ indicates the VD’s position at time *t* and ${\mathrm{move}}_{m}\left(t\right)$ denotes the vector illustrating the VD’s movement during the time slot.

### 3.3. Communication Model

We consider the communication links used to transmit vehicular sensor-data packets from VDs to UAVs and to forward the processed packets from UAVs to the CC. Both the VD–UAV and UAV–CC links are modeled as air-to-ground (A2G) channels. Let $h_{m,k}\left(t\right)$ and $h_{k,c}\left(t\right)$ denote the corresponding horizontal distances. The Euclidean distance between the *m*-th VD and the *k*-th UAV at time *t* and between the *k*-th UAV and the CC are given respectively as follows:

(4) $$\begin{array}{cc} d_{m,k}\left(t\right) & =\sqrt{h_{m,k}^{2}\left(t\right)+H^{2}}, \end{array}$$

(5) $$\begin{array}{cc} d_{k,c}\left(t\right) & =\sqrt{h_{k,c}^{2}\left(t\right)+H^{2}}. \end{array}$$

For a generic A2G link (x→y), the elevation angle in degrees and the line-of-sight (LoS) probability are [39]

(6) $$\vartheta_{x,y}\left(t\right)=\frac{180}{\pi }\arctan\;\left(\frac{H}{h_{x,y}\left(t\right)}\right),\;p_{x,y}^{L}\left(t\right)=\frac{1}{1+a\exp[-b\left(\vartheta_{x,y}\left(t\right)-a\right)]}.$$

Here, *a* and *b* are environment-specific parameters. The average path loss for A2G communication is computed as follows:

(7) $$PL_{x,y}\left(t\right)={\left(\frac{4\pi f_{c}d_{x,y}\left(t\right)}{c}\right)}^{2}\left[p_{x,y}^{L}\left(t\right)10^{\eta^{L}/10}+\left(1-p_{x,y}^{L}\left(t\right)\right)10^{\eta^{\mathrm{NL}}/10}\right],$$

where $f_{c}$ is the carrier frequency; *c* is the speed of light; and $\eta^{L}$ and $\eta^{\mathrm{NL}}$ are the additional LoS and non-line-of-sight (NLoS) losses in dB, respectively. In (7), $PL_{x,y}\left(t\right)$ is expressed as a linear, dimensionless power-loss factor rather than in dB. Consequently, $P_{x}^{\mathrm{tr}}\left(t\right)/PL_{x,y}\left(t\right)$ has units of watts. Since $N_{0}$ is the noise power spectral density in W/Hz, $N_{0}W_{\mathrm{VD}}$ and $N_{0}b_{k}\left(t\right)W$ are the receiver noise powers in watts for the VD–UAV and UAV–CC links, respectively. The interference terms in (11) and (12) are also sums of received powers in watts. Hence, the interference and thermal noise terms in the denominators of (8) and (9) are dimensionally consistent and strictly additive.

The transmission rates for the VD-to-UAV link ($r_{m,k}\left(t\right)$) and the UAV-to-CC link ($r_{k,c}\left(t\right)$) are calculated based on the Shannon formula. They depend on the respective Signal-to-Interference-plus-Noise Ratios (SINRs), which account for interference from other transmitting sources:

(8) $$r_{m,k}^{\mathrm{Sh}}\left(t\right)=W_{\mathrm{VD}}\log_{2}\left(1+\frac{P_{m}^{\mathrm{tr}}\left(t\right)/PL_{m,k}\left(t\right)}{N_{0}W_{\mathrm{VD}}+I_{m,k}\left(t\right)}\right).$$

(9) $$r_{k,c}\left(t\right)=b_{k}\left(t\right)W\log_{2}\left(1+\frac{P_{k}^{\mathrm{tr}}\left(t\right)/PL_{k,c}\left(t\right)}{N_{0}b_{k}\left(t\right)W+I_{k,c}\left(t\right)}\right),$$

where $P_{m}^{\mathrm{tr}}\left(t\right)$ and $P_{k}^{\mathrm{tr}}\left(t\right)$ represent the transmission power of the VD and the UAV, respectively. $N_{0}$ is the noise power spectral density in W/Hz, $W_{VD}$ is the bandwidth allocated for VD-to-UAV communication, and $b_{k}\left(t\right)W$ is the bandwidth assigned to the UAV for communication with the CC.

The physical-layer rate is further subject to the per-slot collection service capability of the UAV sensing interface. Therefore, the effective VD–UAV collection rate used in the packet-level simulator is

(10) $${\tilde{r}}_{m,k}\left(t\right)=\min\left\{r_{m,k}^{\mathrm{Sh}}\left(t\right),\frac{r_{\mathrm{VD}}^{\max}}{T_{s}}\right\},$$

where $r_{\mathrm{VD}}^{\max}=8$ kb/slot is the maximum amount of VD data that can be admitted by one UAV collection link within a slot. Thus, the Shannon expression characterizes the instantaneous physical-layer link capacity, whereas the fixed 8 kb/slot parameter represents a higher-layer collection-service cap. The packet transmission model uses ${\tilde{r}}_{m,k}\left(t\right)$ rather than treating the two rates as independent communication models.

The interferences for the VD–UAV link ($I_{m,k}\left(t\right)$) and the UAV-CC link ($I_{k,c}\left(t\right)$) arise from transmissions of other devices and are calculated as follows:

(11) $$I_{m,k}\left(t\right)=\sum_{\begin{array}{c} m^{\prime }=1\\ m^{\prime }\neq m \end{array}}^{M}\frac{P_{m^{\prime }}^{\mathrm{tr}}\left(t\right)}{PL_{m^{\prime },k}\left(t\right)},$$

(12) $$I_{k,c}\left(t\right)=\sum_{\begin{array}{c} k^{\prime }=1\\ k^{\prime }\neq k \end{array}}^{K}\frac{P_{k^{\prime }}^{\mathrm{tr}}\left(t\right)}{PL_{k^{\prime },c}\left(t\right)}.$$

The total interference is the sum of power from all other transmitting sources, excluding the primary transmitter. This interference term affects the SINR, which, in turn, determines the achievable transmission rates for the VD–UAV and UAV–CC links.

### 3.4. Edge Processing and Offloading Model

The edge processing and offloading model describes how the sensor-data packets generated by the VDs are collected, processed, and forwarded through the UAV edge layer. At each time slot *t*, the *k*-th UAV collects packets from the VDs within its coverage area.

For each sensor-data packet generated by VD *m*, a computation-splitting ratio $d_{m}\left(t\right)\in \left[0,1\right]$ specifies how the packet is processed. Specifically, $d_{m}\left(t\right)$ is the fraction processed locally by the VD, whereas $1-d_{m}\left(t\right)$ is placed in the source buffer for UAV collection. Therefore, the data volumes assigned to local and UAV computation are respectively given by

(13) $$L_{m}^{\mathrm{loc}}\left(t\right)=d_{m}\left(t\right)L_{m}\left(t\right),$$

whereas the UAV-processed portion is

(14) $$L_{m}^{\mathrm{UAV}}\left(t\right)=\left[1-d_{m}\left(t\right)\right]L_{m}\left(t\right).$$

Let $f_{m}^{\mathrm{loc}}\left(t\right)$ denote the effective local data-processing rate of VD *m*. The processing time required for the locally assigned portion is

(15) $$T_{m}^{\mathrm{loc}}\left(t\right)=\frac{L_{m}^{\mathrm{loc}}\left(t\right)}{f_{m}^{\mathrm{loc}}\left(t\right)}=\frac{d_{m}\left(t\right)L_{m}\left(t\right)}{f_{m}^{\mathrm{loc}}\left(t\right)},\;f_{m}^{\mathrm{loc}}\left(t\right)>0.$$

The locally processed portion ($L_{m}^{\mathrm{loc}}\left(t\right)$) is used only by vehicle-side applications and is not transmitted to the UAV or the CC. Therefore, completing local processing does not reset the AoI measured at the CC. Only $L_{m}^{\mathrm{UAV}}\left(t\right)$ enters the UAV collection, processing, and offloading pipeline and can generate a new status update for the CC.

Accordingly, only $L_{m}^{\mathrm{UAV}}\left(t\right)$ enters the UAV collection pipeline. This definition matches the implementation, in which the VD actor outputs the local-computation fraction and the remaining buffered data may be collected by an in-range UAV.

When mobile VD *m* is within the collection range of UAV *k* and sufficient collection-buffer capacity is available, UAV *k* collects $L_{m}^{\mathrm{UAV}}\left(t\right)$ from the VD source buffer. Given the VD–UAV transmission rate (${\tilde{r}}_{m,k}\left(t\right)$), the corresponding collection time is

(16) $$T_{m,k}^{\mathrm{col}}\left(t\right)=\frac{L_{m}^{\mathrm{UAV}}\left(t\right)}{{\tilde{r}}_{m,k}\left(t\right)}=\frac{\left[1-d_{m}\left(t\right)\right]L_{m}\left(t\right)}{{\tilde{r}}_{m,k}\left(t\right)},\;{\tilde{r}}_{m,k}\left(t\right)>0.$$

During each time slot (*t*), the *k*-th UAV dedicates computational resources to unprocessed data in its collection buffer, represented as $D_{k}^{\mathrm{col}}$. The processing decision can be expressed as

(17) $${\mathrm{exec}}_{k}\left(t\right)=\left[{\mathrm{exec}}_{k}^{0}\left(t\right),{\mathrm{exec}}_{k}^{1}\left(t\right),\ldots ,{\mathrm{exec}}_{k}^{B_{k}^{\mathrm{col}}\left(t\right)}\left(t\right)\right],$$

where ${\mathrm{exec}}_{k}^{0}\left(t\right)=1$ denotes the no-operation action, i.e., no packet is selected from the collection buffer for execution in time slot *t*. For i≥1, ${\mathrm{exec}}_{k}^{i}\left(t\right)=1$ indicates that the *i*-th valid packet is selected.

(18) $$\sum_{i=0}^{B_{k}^{\mathrm{col}}\left(t\right)}{\mathrm{exec}}_{k}^{i}\left(t\right)=1,\;{\mathrm{exec}}_{k}^{i}\left(t\right)\in \left\{0,1\right\}.$$

Let $L_{k,i}^{\mathrm{col}}\left(t\right)$ denote the size of the *i*-th data block in the collection buffer. The total data volume selected for processing by UAV *k* is

(19) $$L_{k}^{\mathrm{proc}}\left(t\right)=\sum_{i=1}^{B_{k}^{\mathrm{col}}\left(t\right)}{\mathrm{exec}}_{k}^{i}\left(t\right)L_{k,i}^{\mathrm{col}}\left(t\right).$$

The required UAV processing time is given by

(20) $$T_{k}^{\mathrm{exe}}\left(t\right)=\frac{L_{k}^{\mathrm{proc}}\left(t\right)}{f_{k}^{c}\left(t\right)}=\frac{\sum_{i=1}^{B_{k}^{\mathrm{col}}\left(t\right)}{\mathrm{exec}}_{k}^{i}\left(t\right)L_{k,i}^{\mathrm{col}}\left(t\right)}{f_{k}^{c}\left(t\right)},$$

where $f_{k}^{c}\left(t\right)$ denotes the effective data-processing rate of UAV *k*.

Data processed by the UAVs is temporarily stored in an executed buffer ($D_{k}^{\mathrm{exe}}\left(t\right)$) before being transferred to the CC. At time slot *t*, the *k*-th UAV assesses its executed buffer and determines the dataset for offloading, referred to as the offloading decision, which can be expressed as

(21) $${\mathrm{off}}_{k}\left(t\right)=\left[{\mathrm{off}}_{k}^{0}\left(t\right),{\mathrm{off}}_{k}^{1}\left(t\right),\ldots ,{\mathrm{off}}_{k}^{B_{k}^{\mathrm{exe}}\left(t\right)}\left(t\right)\right],$$

where ${\mathrm{off}}_{k}^{0}\left(t\right)=1$ represents the no-operation action, meaning that no packet is offloaded from the executed buffer in time slot *t*. For i≥1, ${\mathrm{off}}_{k}^{i}\left(t\right)=1$ indicates that the *i*-th valid executed packet is selected for transmission to the CC.

(22) $$\sum_{i=0}^{B_{k}^{\mathrm{exe}}\left(t\right)}{\mathrm{off}}_{k}^{i}\left(t\right)=1,\;{\mathrm{off}}_{k}^{i}\left(t\right)\in \left\{0,1\right\}.$$

Let $L_{k,i}^{\mathrm{exe}}\left(t\right)$ denote the size of the *i*-th data block in the executed buffer. The total data volume selected for offloading is

(23) $$L_{k}^{\mathrm{off}}\left(t\right)=\sum_{i=1}^{B_{k}^{\mathrm{exe}}\left(t\right)}{\mathrm{off}}_{k}^{i}\left(t\right)L_{k,i}^{\mathrm{exe}}\left(t\right).$$

Based on the UAV–CC transmission rate ($r_{k,c}\left(t\right)$), the corresponding offloading time is calculated as

(24) $$T_{k,c}^{\mathrm{off}}\left(t\right)=\frac{L_{k}^{\mathrm{off}}\left(t\right)}{r_{k,c}\left(t\right)}=\frac{\sum_{i=1}^{B_{k}^{\mathrm{exe}}\left(t\right)}{\mathrm{off}}_{k}^{i}\left(t\right)L_{k,i}^{\mathrm{exe}}\left(t\right)}{r_{k,c}\left(t\right)},\;r_{k,c}\left(t\right)>0.$$

The CC receives the packets that have already been processed by the UAVs and performs bandwidth allocation and system coordination. Therefore, no additional CC-side computation time is considered. The above quantities represent transmission and processing service times, whereas any additional waiting time is captured by the evolution of the VD and UAV buffers.

### 3.5. Problem Formulation

For real-time vehicular sensing applications, the usefulness of a sensor-data packet decreases as the elapsed time since its generation increases. Therefore, the AoI is used to quantify the freshness of the vehicular sensing information available at the CC. At time slot *t*, the AoI associated with VD *m* is defined as

(25) $$\Delta_{m}\left(t\right)=t-T_{g}^{m}\left(t\right),$$

where $T_{g}^{m}\left(t\right)$ denotes the generation timestamp of the most recent sensor-data packet from the *m*-th VD that has been successfully processed and received by the CC. This packet represents the latest available status update from the corresponding VD.

To characterize the overall freshness of vehicular sensing information, the average AoI across all VDs is calculated as

(26) $$\hat{\Delta }\left(t\right)=\frac{1}{M}\sum_{m=1}^{M}\Delta_{m}\left(t\right),$$

where *M* is the total number of vehicular sensing sources. A lower value of $\hat{\Delta }\left(t\right)$ indicates that the CC has received more recent sensor data from the VDs.

The objective is to minimize the average AoI of vehicular sensing in the three-layer UAV-assisted edge network. Accordingly, the optimization problem (P) is formulated as follows:

(27) $$\begin{array}{cc} \; & P:\min_{\left\{d_{m}\left(t\right),\;{\mathrm{move}}_{k}\left(t\right),\;{\mathrm{exec}}_{k}\left(t\right),\;{\mathrm{off}}_{k}\left(t\right),\;b_{k}\left(t\right)\right\}}\hat{\Delta }\left(t\right)\\ \; & \mathrm{subject}\;\mathrm{to}:\\ \; & C1:\;{∥{\mathrm{move}}_{k}\left(t\right)∥}_{2}\leq r_{\mathrm{move}}^{k},\;\forall k=1,\ldots ,K,\\ \; & C2:\;\sum_{i=0}^{B_{k}^{\mathrm{col}}\left(t\right)}{\mathrm{exec}}_{k}^{i}\left(t\right)=1,\;\forall k=1,\ldots ,K,\\ \; & C3:\;\sum_{i=0}^{B_{k}^{\mathrm{exe}}\left(t\right)}{\mathrm{off}}_{k}^{i}\left(t\right)=1,\;\forall k=1,\ldots ,K,\\ \; & C4:\;0\leq d_{m}\left(t\right)\leq 1,\;\forall m=1,\ldots ,M,\\ \; & C5:\;{\mathrm{exec}}_{k}^{i}\left(t\right),{\mathrm{off}}_{k}^{j}\left(t\right)\in \left\{0,1\right\},\;i=0,\ldots ,B_{k}^{\mathrm{col}}\left(t\right),\;j=0,\ldots ,B_{k}^{\mathrm{exe}}\left(t\right),\\ \; & C6:\;b_{k}\left(t\right)\geq 0,\;\sum_{k=1}^{K}b_{k}\left(t\right)\leq 1,\;\forall k=1,\ldots ,K,\\ \; & C7:\;p_{k}\left(t\right)\in \Omega ,\;\forall k=1,\ldots ,K.\\ \; & C8:\;0\leq Q_{m}^{\mathrm{src}}\left(t\right)\leq B_{m,\max},\;\forall m=1,\ldots ,M.\\ \; & C9:\;0\leq D_{k}^{\mathrm{col}}\left(t\right)\leq B_{k,\max}^{\mathrm{col}},\;0\leq D_{k}^{\mathrm{exe}}\left(t\right)\leq B_{k,\max}^{\mathrm{exe}},\;\forall k=1,\ldots ,K. \end{array}$$

In this formulation, $\{d_{m}\left(t\right)$, ${\mathrm{move}}_{k}\left(t\right)$, ${\mathrm{exec}}_{k}\left(t\right)$, ${\mathrm{off}}_{k}\left(t\right)$, and $b_{k}\left(t\right)\}$ denote the control variables of the VDs, UAVs, and the CC. Specifically, $d_{m}\left(t\right)$ is the computation-splitting ratio of VD *m*, which specifies the fractions of the generated sensor data assigned to UAV-side edge processing and local computation. The ${\mathrm{move}}_{k}\left(t\right)$ variable specifies the movement of UAV *k*. The ${\mathrm{exec}}_{k}\left(t\right)$ and ${\mathrm{off}}_{k}\left(t\right)$ vectors specify the packets selected for UAV-side processing and subsequent offloading to the CC, respectively, while $b_{k}\left(t\right)$ denotes the fraction of the total UAV–CC bandwidth assigned to UAV *k*.

Constraint C1 limits the displacement of each UAV within one time slot. Constraints C2 and C3 ensure that each UAV selects, at most, one available packet for processing and offloading, respectively. Constraint C4 defines the feasible range of the partial computation-offloading ratio, where $d_{m}\left(t\right)=1$ corresponds to fully local processing and $d_{m}\left(t\right)=0$ corresponds to fully UAV-assisted processing. Constraint C5 specifies the binary domains of the UAV processing and offloading decisions. Constraint C6 guarantees a feasible bandwidth allocation among the UAVs, while C7 confines each UAV to the predefined operating region (Ω). Constraints C8 and C9 specify the finite source-buffer, collection-buffer, and executed-buffer capacities.

Several modeling assumptions are adopted to keep the optimization focused on network-level sensing freshness. The UAV altitude is fixed at *H*, while only horizontal mobility is optimized. UAV propulsion energy is not included as an optimization variable, and sufficient battery capacity is assumed over the considered sensing horizon. Interference is determined in each time slot from the concurrently active transmitters and the corresponding link conditions. Transmission failures are reflected through the achievable communication rate and packet-buffer evolution. Moreover, AoI is updated only when a processed sensor-data packet is successfully received by the CC; therefore, the time spent in collection, UAV-side processing, buffering, and offloading is implicitly accumulated in the final information age.

## 4. Significance-Aware Heterogeneous Federated Actor–Critic Framework

**Figure 2:**
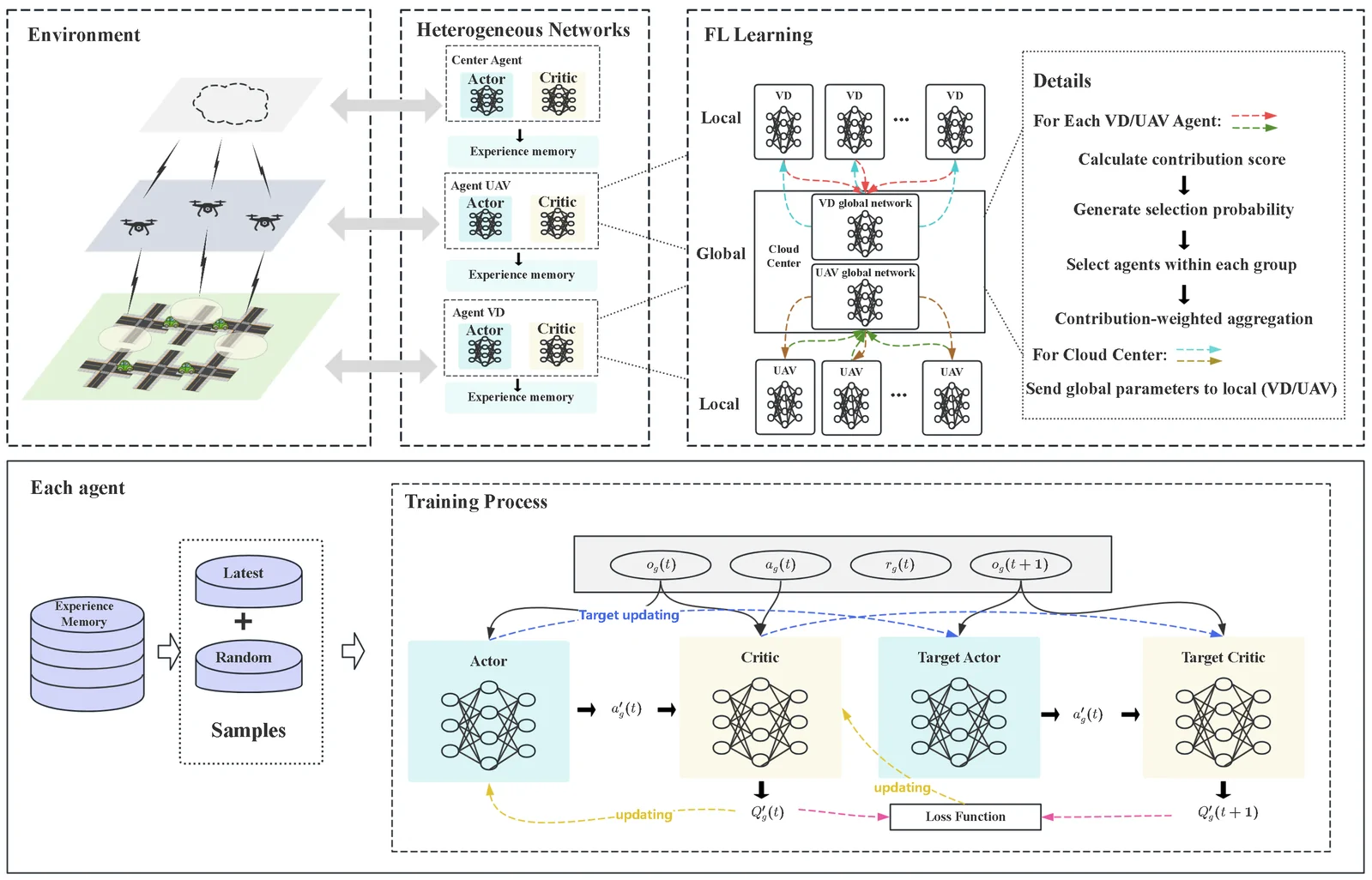
Architecture of CHFAC. In the FL Learning module, the red and green dashed arrows represent the contribution calculation, agent selection, and contribution-weighted aggregation process, while the cyan and brown dashed arrows denote the transmission of global parameters from the cloud center to local agents. In the Training Process module, the black solid arrows represent the data flow, the blue dashed arrows indicate the soft update of target networks, the yellow dashed arrows show the parameter updates for both Actor and Critic networks, and the purple dashed arrows represent the loss calculation for Actor and Critic.

To solve the optimization problem P, we frame it as a decentralized partially observable Markov decision process (DEC-POMDP) and adopt a reinforcement learning (RL) methodology. Although value-based RL methods can estimate action utilities effectively, they encounter difficulties in large and complex action spaces due to the high computational burden. On the other hand, policy-based methods like deep deterministic policy gradient (DDPG) and advantage actor–critic (A2C) offer advantages by directly optimizing actions, which simplifies decision-making in expansive action spaces. Nonetheless, traditional single-agent or centralized approaches become infeasible in multi-agent environments because of scalability issues and the complex parameter management required for the coordination of multiple agents [29]. To address these challenges, we propose the CHFAC framework, leveraging FL principles to improve multi-agent cooperation and system efficiency. The CHFAC framework, illustrated in Figure 2, fosters the development of a novel algorithm that enhances AoI performance across the UAV-assisted edge architecture.

**Figure 3:**
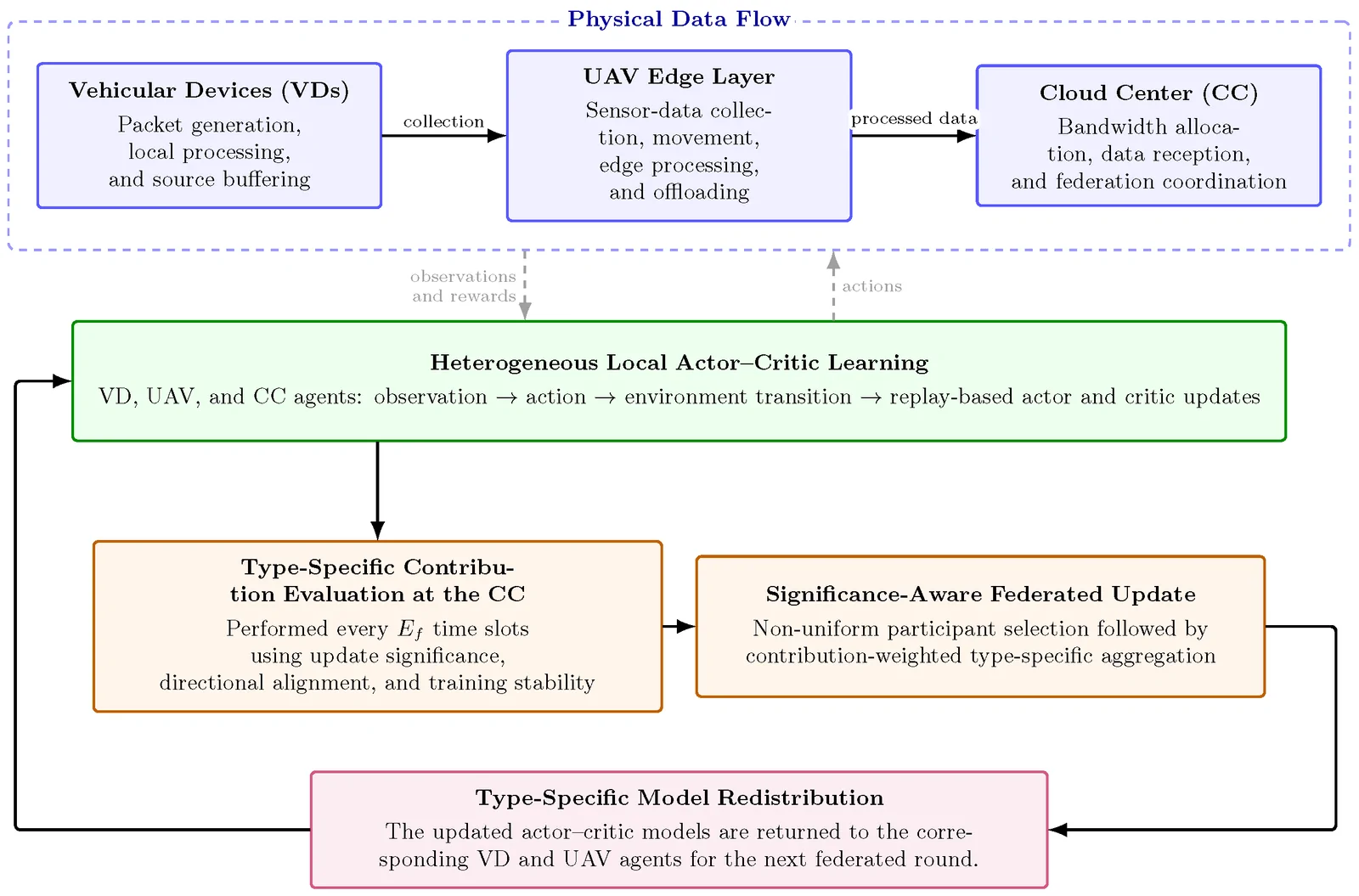
Information and learning flow of CHFAC. The upper layer (blue background) shows vehicular sensing-data transmission and processing; the middle layer (green background) presents heterogeneous local actor-critic learning; and the lower layers (orange and pink backgrounds) illustrate type-specific contribution evaluation, significance-aware participant selection, weighted aggregation, and model redistribution.

Figure 3 provides an overview of the physical data processing and collaborative learning procedures in CHFAC. VDs, UAVs, and the CC interact with the environment and perform heterogeneous local actor–critic updates. Every $E_{f}$ time slots, the CC evaluates the type-specific VD and UAV updates, selects informative participants, aggregates the selected actor–critic models, and redistributes the updated models.

### 4.1. DEC-POMDP Formulation for Cooperative Vehicular Sensing

Our enhanced Decentralized Partially Observable Markov Decision Process (DEC-POMDP) model consists of a set of heterogeneous agents (G=M∪K∪{c}) comprising *M* VDs, *K* UAVs, and one CC. These agents interact with their environment based on their own local observations. The system’s state space is denoted as S, and the individual observation spaces for each agent are represented as ${\left\{O_{g}\right\}}_{g\in G}$. Each agent is associated with its own action set (${\left\{A_{g}\right\}}_{g\in G}$) and operates under a reward mechanism (R), with decisions driven by partial observations (${\left\{O_{g}\right\}}_{g\in G}$) according to each agent’s policy. This model is particularly suited for the IoV-MEC system, which involves dynamic decision-making under partial observability and uncertainty.

#### 4.1.1. State and Observation Representation

Each agent’s state captures both spatial and operational information. At any given time (*t*), the system’s overall state (S) incorporates the positions and resource usage details of all VDs, as well as the positions, buffer statuses, and bandwidth allocation statuses of all UAVs:

(28) $$\begin{array}{cc} S(t)= & \left\{\left(p_{m}\left(t\right),z_{m}\left(t\right)\right)|m\in M\right\}\\ \; & \cup \{\left(p_{k}\left(t\right),b_{k}\left(t\right),z_{k}\left(t\right)\right)|k\in K\}. \end{array}$$

Here, M represents the set of VDs, while K refers to the set of UAVs in the system. $p_{m}\left(t\right)$ is the position, and $z_{m}\left(t\right)$ indicates the buffer state of VD *m*. $p_{k}\left(t\right)$ represents the position, $b_{k}\left(t\right)$ is the allocated bandwidth fraction, and $z_{k}\left(t\right)$ the buffer status of UAV *k*. The $Cov_{k}\left(t\right)$ set refers to the VDs covered by UAV *k*, and $\left\{p_{m}\left(t\right)\right\}$ and ${\left\{z_{m}\left(t\right)\right\}}_{m\in Cov_{k}\left(t\right)}$ describe the positions and buffer statuses of these VDs.

Each agent observes only a limited subset of the system’s state. The observation space for each agent type is defined as follows:

- VDs: For a VD, the observation at time *t* consists of its current position and buffer status:
  (29) $$O_{m}\left(t\right)=\left(p_{m}\left(t\right),z_{m}\left(t\right)\right)$$
- UAVs: Each UAV observes not only its own state but also the states of VDs within its coverage area:
  (30) $$\begin{array}{c} O_{k}\left(t\right)=\left(p_{k}\left(t\right),b_{k}\left(t\right),z_{k}\left(t\right),{\left\{p_{m}\left(t\right)\right\}}_{m\in Cov_{k}\left(t\right)},{\left\{z_{m}\left(t\right)\right\}}_{m\in Cov_{k}\left(t\right)}\right) \end{array}$$
- CC: The CC has a more comprehensive view, aggregating the states of all UAVs:
  (31) $$O_{\mathrm{CC}}\left(t\right)=\underset{k\in K}{⋃}\left(p_{k}\left(t\right),b_{k}\left(t\right),z_{k}\left(t\right)\right)$$
  Because the CC model is not federated with the VD or UAV models, the type-specific global models do not directly enter $O_{\mathrm{CC}}\left(t\right)$. Their effects are reflected through the UAV states generated by the federated VD/UAV policies, particularly the UAV positions and buffer occupancies used by the CC actor for bandwidth allocation.

#### 4.1.2. Action Spaces and System Operations

The action space of each heterogeneous agent is defined consistently with the feasible domain of Problem P in (27). In particular, every action generated by an actor is mapped to the feasible set specified by Constraints C1–C9 before being executed in the environment.

VDs: VD *m* outputs the continuous local-computation fraction:

(32) $$a_{m}\left(t\right)=\left\{d_{m}\left(t\right)\right\},\;0\leq d_{m}\left(t\right)\leq 1,$$

which directly satisfies Constraint C4.

UAVs: The action of UAV *k* contains its movement, packet-execution decision, and packet-offloading decision:

(33) $$a_{k}\left(t\right)=\left\{{\mathrm{move}}_{k}\left(t\right),{\mathrm{exec}}_{k}\left(t\right),{\mathrm{off}}_{k}\left(t\right)\right\}.$$

The movement component is projected onto the feasible displacement and operating-region constraints (C1 and C7). The execution and offloading components satisfy C2, C3, and C5. Both discrete heads include an explicit no-operation category indexed by zero. Thus, ${\mathrm{exec}}_{k}^{0}\left(t\right)=1$ means that no collected packet is executed, whereas ${\mathrm{off}}_{k}^{0}\left(t\right)=1$ means that no executed packet is offloaded in the current slot. Invalid packet indices caused by empty buffer positions are removed by feasibility masks. Constraints C8 and C9 are enforced by rejecting actions that would exceed the corresponding buffer capacities.

CC: The CC generates the bandwidth-allocation vector:

(34) $$a_{\mathrm{CC}}\left(t\right)=b\left(t\right)=\left[b_{1}\left(t\right),\ldots ,b_{K}\left(t\right)\right],$$

subject to

(35) $$b_{k}\left(t\right)\geq 0,\;\sum_{k=1}^{K}b_{k}\left(t\right)\leq 1,$$

as required by Constraint C6. The CC actor uses a Softmax-based output mapping, followed by the feasible bandwidth mapping, to ensure that the executed bandwidth vector remains within the domain of Problem P.

#### 4.1.3. State Transition and Reward Mechanism

The state transition function (P(S(t+1)∣S(t),A(t))) specifies the conditional distribution of the next system state, given the current state and the executed joint action. The reward ($r_{g}\left(t\right)$) for each agent is derived from the system’s AoI performance at time *t*:

(36) $$r_{g}\left(t\right)=-\hat{\Delta }\left(t\right)\;\mathrm{for}\;g\in G.$$

To encourage optimal long-term decisions, we define a cumulative reward function with a discount factor:

(37) $$R_{g}\left(t\right)=-\sum_{i=0}^{T}\gamma^{i}\cdot \hat{\Delta }\left(t+i\right),$$

where γ is the discount factor and *T* is the length of the evaluation window. This approach incentivizes agents to consider the long-term impact of their actions in terms of minimizing the system’s AoI, thereby improving the overall performance.

### 4.2. CHFAC Framework Design

In the CHFAC framework, each agent is equipped with its own actor and critic neural networks, allowing it to independently interact with the environment and learn optimal strategies tailored to its specific role. The strength of the CHFAC framework lies in its ability to leverage heterogeneity, meaning that the neural network architectures are customized for each agent type—VDs, UAVs, and CC—based on their operational roles within the system. Additionally, FL techniques are employed to ensure that these agents collaborate effectively while synchronizing type-specific model updates within the VD and UAV federation groups.

Neural Network Architecture: In the CHFAC framework, the networks are crucial in helping the agents make informed decisions that lead to the optimization of their respective operational objectives.

VD Actor Network and Critic Network Design:

- The VD actor network outputs the continuous computation-splitting ratio ($d_{m}\left(t\right)$). It adopts a multi-layer perceptron (MLP) to process the local observation and produces a scalar output that is mapped to [0,1]. The resulting value specifies the fraction of the current sensor-data packet processed locally by the VD; the remaining fraction enters the UAV collection pipeline.
- The corresponding critic estimates the expected cumulative reward of the selected splitting ratio under the current observation. Since the reward is defined as the negative AoI value, a larger critic output indicates a policy with better information freshness performance. The network is intentionally lightweight because its input is the local buffer state. The VD critic takes the same observation and the selected fraction as input and outputs an action-value estimate, which represents the expected discounted cumulative reward under the current policy.

UAV Actor Network and Critic Network Design:

- The UAV actor network handles more complex tasks, combining both spatial and resource management decisions. It employs a convolutional neural network (CNN) to process the spatial data, such as UAV position and trajectory information, and uses an MLP to handle resource management, including buffer and bandwidth data. This hybrid approach allows the UAV to efficiently manage its movement while ensuring optimal resource allocation.
- The UAV critic network receives a comprehensive set of inputs, including spatial maps, buffer statuses, movement actions, and bandwidth settings. The network utilizes a CNN for analysis of spatial relationships and an MLP to integrate other operational data. This structure enables the UAV to accurately evaluate the potential rewards of its actions, leading to more informed decision-making.

CC Actor Network and Critic Network Design:

- The CC actor network focuses on optimizing bandwidth allocation across the UAVs. It uses an MLP to process information about the buffer statuses of the UAVs and their positional data, determining how to distribute bandwidth efficiently to maximize system performance.
- The CC critic network evaluates the results of the bandwidth allocation decisions made by the actor network. As input, it takes the buffer statuses, positions, and current bandwidth allocations of all UAVs. Dense layers are employed to assess how effectively the allocated bandwidth meets system requirements, providing valuable feedback for future bandwidth allocation decisions.

Each neural network in the CHFAC framework is designed to optimize actions based on real-time data input, enabling agents to make better decisions in a collaborative and dynamic environment. By tailoring the architecture of the actor and critic networks to each agent’s role, the CHFAC framework enhances the overall efficiency and decision-making process in the system, ultimately leading to more effective multi-agent cooperation and improved system performance.

The heterogeneity of CHFAC arises from the role-specific actor–critic architectures adopted by the VDs, UAVs, and the CC. Since agents of different types have different parameter spaces, cross-type parameter averaging is not performed. Instead, federated aggregation is conducted separately within the VD and UAV groups. All agents belonging to the same group use identical actor and critic architectures and therefore have parameter vectors with identical dimensions.

The concatenated online actor and critic parameters of agent *i* in federation group *q* are denoted by

(38) $$\vartheta_{q,i}=\left[\begin{array}{c} \theta_{q,i}\\ \phi_{q,i} \end{array}\right]\in R^{P_{q}},\;q\in \left\{\mathrm{VD},\mathrm{UAV}\right\},$$

where $P_{q}$ is the total number of online actor–critic parameters for agents of type *q*. Both the actor and critic parameters are uploaded and aggregated within their corresponding type-specific groups. The CC maintains its own local actor–critic model independently and serves only as the coordinator of the federated procedure. Its actor and critic parameters are never included in either the VD-side or UAV-side parameter averaging. Accordingly, federated aggregation is performed only within q∈{VD,UAV}, whereas the CC actor–critic model is updated exclusively from its own replay buffer and local actor–critic losses.

The influence of the aggregated VD and UAV models on the CC is therefore indirect rather than parameter-level. After each federated round, the updated type-specific models are redistributed to the corresponding VD and UAV agents. These models affect their subsequent computation, movement, execution, and offloading actions, which, in turn, change the UAV positions and buffer states observed by the CC. The CC actor then uses these updated system states to determine the bandwidth-allocation vector. Thus, the CC coordinates federated learning while preserving an independent local decision model for bandwidth allocation. The target actor and critic networks are not directly aggregated; they continue to track the received online networks through the soft-update rule.

Table 3 summarizes the actor and critic network architectures of the heterogeneous agents in CHFAC. Here, $N_{x}^{s}\times N_{y}^{s}\times C_{s}$ denotes the UAV spatial-observation tensor, while $N_{x}^{a}\times N_{y}^{a}$ denotes the UAV movement-action map. Agents belonging to the same role use identical actor and critic architectures. Therefore, type-wise federated aggregation is performed over complete actor–critic parameter vectors with compatible dimensions. Invalid execution and offloading entries are removed using feasibility-aware action masks. When no valid packet is available, a no-operation action is applied. Each target network has the same architecture as its corresponding online network.

For the UAV execution and offloading heads, the additional output indexed by zero represents the no-operation action. The remaining outputs correspond to valid packet positions in the respective buffers. A feasibility mask is applied before categorical selection so that empty or invalid buffer entries cannot be selected.

#### 4.2.1. Enhancements with Target Networks

To stabilize training in temporal-difference learning, we incorporate target networks for both the actor and critic. These target networks mirror the architecture of the main actor and critic networks but are updated at a slower rate to avoid instability. The target network parameters are updated incrementally using a soft update rule as follows:

(39) $${\theta_{g}^{\prime }}^{\;t+1}=\tau \theta_{g}^{t}+\left(1-\tau \right){\theta_{g}^{\prime }}^{\;t}.$$

(40) $${\phi_{g}^{\prime }}^{\;t+1}=\tau \phi_{g}^{t}+\left(1-\tau \right){\phi_{g}^{\prime }}^{\;t}.$$

where τ is the update coefficient, controlling the rate of change. These updates are applied every $T_{\mathrm{up}}=8$ interaction slots, ensuring a smoother learning process and improving convergence.

#### 4.2.2. Distributed Online Training Execution

Having defined the feasible action domains in Section 4.1.2 and the role-specific actor–critic architectures, we now describe how these networks are trained online and how the resulting local policies are combined through federation. This subsection presents the online training–execution procedure; the following subsection details the contribution-aware aggregation that uses the trained local updates. The framework operates online: each agent executes an action, observes the next state and the corresponding reward, stores the transition, and updates its local networks. Since the reward is defined as the negative AoI value, higher cumulative rewards indicate fresher information states. The VD fraction and CC bandwidth vector are continuous. UAV displacement, packet execution, and packet offloading are discrete. The actor output is therefore passed through a type-specific feasibility map ($\Pi_{g}$): sigmoid and Softmax mappings are used for continuous constrained outputs, whereas the largest actor logit is selected for each discrete head.

Exploration acts on the actor output rather than on an argmax of the critic. At training time, the executed action is

(41) $$a_{g}\left(t\right)=\left\{\begin{array}{cc} {\tilde{a}}_{g}\left(t\right)∼\mathrm{Unif}\left(A_{g}\right),\; & \mathrm{with}\;\mathrm{probability}\;\epsilon ,\\ \Pi_{g}\;\left(A_{g}\left(O_{g}\left(t\right);\theta_{g}\right)\right),\; & \mathrm{with}\;\mathrm{probability}\;1-\epsilon . \end{array}\right.$$

The initial exploration probability is 0.2. It is multiplied by 0.9 after each 200-slot interaction window and is lower-bounded by 0.05. Evaluation uses the deterministic actor output without random replacement. During training, exploration noise is added to continuous actor outputs, while the deterministic actor policy is directly adopted during testing.

Throughout the learning process, agents store transitions in an experience buffer, which is used to update the networks. The critic network is trained by minimizing the temporal-difference loss function $\ell_{C}$, defined as

(42) $$\ell_{C}\left(\phi_{g}\right)=E\left[{\left(Q_{g}\left(O_{g}\left(t\right),a_{g}\left(t\right);\phi_{g}\right)-y_{g}\left(t\right)\right)}^{2}\right].$$

The target value is calculated based on the immediate reward and the target critic estimation of the next state:

(43) $$y_{g}\left(t\right)=r_{g}\left(t\right)+\gamma Q_{g}^{\prime }\left(O_{g}\left(t+1\right),A_{g}^{\prime }\left(O_{g}\left(t+1\right);\theta_{g}^{\prime }\right);\phi_{g}^{\prime }\right),$$

where γ denotes the reward discount factor and $Q_{g}^{\prime }$ and $A_{g}^{\prime }$ represent the target critic and target actor networks, respectively. This target formulation improves the stability of critic training by reducing the correlation between the current estimation and the target value.

The actor network is optimized by maximizing the expected cumulative reward estimated by the critic. Therefore, the actor loss is defined as the negative critic value:

(44) $$\ell_{A}\left(\theta_{g}\right)=-E\left[Q_{g}\left(O_{g}\left(t\right),A_{g}\left(O_{g}\left(t\right);\theta_{g}\right);\phi_{g}\right)\right].$$

Minimizing $\ell_{A}\left(\theta_{g}\right)$ is equivalent to maximizing the critic-estimated cumulative reward. Since the reward is defined as the negative AoI value, the actor update naturally guides the policy toward actions that reduce the long-term average AoI.

Both actor and critic networks are updated using gradient-based optimization with the Adam optimizer. The parameter update can be generally expressed as

(45) $$\phi_{g}^{t+1}=\phi_{g}^{t}-\eta_{C}\nabla_{\phi_{g}}\ell_{C}\left(\phi_{g}\right).$$

(46) $$\theta_{g}^{t+1}=\theta_{g}^{t}-\eta_{A}\nabla_{\theta_{g}}\ell_{A}\left(\theta_{g}\right).$$

where $\eta_{C}$ and $\eta_{A}$ are the learning rates for the critic and actor networks, respectively.

#### 4.2.3. Significance-Aware FL with Contribution-Aware Aggregation

Federated updating is performed separately for the VD and UAV groups. At each federated round, agents first complete their local actor–critic updates. The CC then evaluates the candidate updates using three quantities: the normalized update-significance metric, directional alignment with the current group learning direction, and a stability penalty. These quantities are used to determine participant-selection probabilities and aggregation weights.

The procedure contains four sequential stages. First, each agent performs a proximal-regularized local update. Second, the CC evaluates the significance and directional consistency of the candidate update. Third, a non-negative Softmax transformation is applied to obtain the participant selection probability. Finally, only the selected updates are aggregated within the corresponding agent type and redistributed. The CC model, itself, does not participate in this averaging process. The process, as detailed below, relies on the notation provided in Table 4.

At the start of each global round (*t*), each agent ($i\in N_{q}$) receives the type-specific global model ($\vartheta_{q}^{t}={\left[{\left(\theta_{q}^{t}\right)}^{⊤},{\left(\phi_{q}^{t}\right)}^{⊤}\right]}^{⊤}$). To stabilize local training, we employ a regularized objective function ($H_{i}$), which combines the agent’s local loss ($F_{i}$) with a proximal term:

(47) $$H_{i}\left(\vartheta ;\vartheta_{q}^{t}\right)=F_{i}\left(\vartheta \right)+\frac{\mu }{2}{∥\vartheta -\vartheta_{q}^{t}∥}_{2}^{2},$$

where μ is a regularization hyperparameter. Agent *i* then performs local updates to obtain its new parameters ($\vartheta_{q,i}^{t+1}$).

After local training, the CC evaluates each agent’s contribution based on two key metrics derived from the agents’ uploaded information.

1. Update Significance: ($\delta_{i}^{t}$)
  This metric quantifies how much an agent’s model has changed. It is calculated by normalizing the magnitude of the agent’s effective update step by the magnitude of its current-round gradient. Based on our implementation, it is formulated as
  (48) $$\delta_{i}^{t}=\frac{{∥\nabla F_{i}\left(\vartheta_{q}^{t}\right)+\mu \left(\vartheta_{q,i}^{t+1}-\vartheta_{q}^{t}\right)∥}_{2}}{{∥g_{i}^{t}∥}_{2}+\epsilon_{s}}=\frac{∥g_{i}^{t}+\mu \left(\vartheta_{q,i}^{t+1}-\vartheta_{q}^{t}\right){∥}_{2}}{{∥g_{i}^{t}∥}_{2}+\;\epsilon_{s}},$$
  where $\epsilon_{s}>0$ prevents division by zero, $g_{i}^{t}$ is the local gradient at the start of round *t*, and $g_{i}^{t-1}$ is the local gradient from round t−1. This captures the volatility and scale of an agent’s learning dynamics.
2. Contribution Score ($z_{i}^{t}$):
  The final contribution score, $z_{i}^{t}$, assesses the quality of an agent’s update. It measures the alignment between the current global learning direction and the agent’s historical update direction while penalizing unstable agents. The global direction is approximated by the average gradient, i.e., $\bar{g_{q}^{t}}=\frac{1}{N_{q}}\sum_{j\in N_{q}}g_{j}^{t}$. The score is then
  (49) $$z_{i}^{t}={\left(\bar{g_{q}^{t}}\right)}^{⊤}g_{i}^{t-1}-\psi \delta_{i}^{t}{∥\bar{g_{q}^{t}}∥}_{2}^{2}.$$
  Here, the dot product (${\left(\bar{g_{q}^{t}}\right)}^{⊤}g_{i}^{t-1}$) rewards agents whose past updates are consistent with the current global objective. The second term, controlled by hyperparameter ψ, penalizes excessively large update magnitudes, thereby promoting stable model updates. The calculated contribution scores guide the final aggregation step. Accordingly, the contribution score rewards directional consistency while discouraging excessively large and potentially unstable model changes.
3. Selection Probability ($p_{i}^{t}$):
  Signed raw scores are not used directly as probabilities or aggregation weights. Instead, a numerically stable exponential transformation gives
  (50) $$I_{i}^{t}=\exp\;\left[\beta \left(z_{i}^{t}-\max_{j\in N_{q}}z_{j}^{t}\right)\right],$$
  followed by
  (51) $$p_{i}^{t}=\frac{I_{i}^{t}}{\sum_{j\in N_{q}}I_{j}^{t}}.$$
  The subtraction of the group maximum prevents overflow without changing the normalized probabilities. The Softmax coefficient (β) controls how strongly selection favors high-scoring agents. The CC samples $S_{q}^{t}$ agents without replacement according to $\left\{p_{i}^{t}\right\}$. The softmax normalization ensures that the transformed contribution scores remain non-negative probabilities, even when the raw scores are negative.
4. Weighted Global Update:
  For federation group *q*, the CC samples $S_{q}$ agents and obtains the selected set ($S_{q}^{t}$) according to the probabilities ($\left\{p_{i}^{t}\right\}$). The global model is updated by aggregating the parameter changes of the selected agents. The aggregation weights are computed from the non-negative Softmax-transformed contribution values ($I_{i}^{t}$) rather than from the signed raw scores ($z_{i}^{t}$).
  (52) $$\vartheta_{q}^{t+1}=\vartheta_{q}^{t}+\sum_{i\in S_{q}^{t}}\left(\frac{I_{i}^{t}}{\sum_{j\in S_{q}^{t}}I_{j}^{t}}\right)\left(\vartheta_{q,i}^{t+1}-\vartheta_{q}^{t}\right)$$
  This contribution-aware selection and weighted aggregation process enhances learning efficiency. The new global model ($\vartheta_{q}^{t+1}$) is then sent back to the agents for the next round.

The proposed CHFAC framework is summarized in Algorithm 1.
**Algorithm 1** Revised Online CHFAC Framework  1:**Initialization:**  2:Set up system parameters and initialize neural network parameters $\theta_{m},\theta_{k},\theta_{c},\phi_{m},\phi_{k},\phi_{c}$.  3:Initialize experience buffer $B_{g}$.  4:**for** epoch=1 to MAXEPOCH **do**  5:      **for** each agent g∈G **do**  6:            Record observation $O_{g}\left(t\right)$ at time *t*.  7:            Generate $a_{g}\left(t\right)$ according to the actor policy and the exploration mechanism.  8:            Apply the feasibility mapping defined by C1–C9; use the index-zero            no-operation action for UAV execution/offloading when applicable.  9:            Execute action $a_{g}\left(t\right)$ in the environment.10:            Save tuple $\left\{O_{g}\left(t\right),a_{g}\left(t\right),r_{g}\left(t\right),O_{g}\left(t+1\right)\right\}$ to $B_{g}$.11:            Extract a batch from buffer $B_{g}$.12:            Select actions for future time steps and evaluate Q-values.13:            Compute loss terms $\ell_{C}\left(\phi_{g}\right)$ and $\ell_{A}\left(\theta_{g}\right)$ using (42) and (44).14:            Update critic and actor networks via Adam optimizer (45), (46).15:            **if** $t\;\mathrm{mod}\;T_{\mathrm{up}}==0$ **then**16:                  Update target networks according to (39) and (40).17:            **end if**18:            **if** $t\;\mathrm{mod}\;E_{f}=0$ **then**19:                  **for** each federated agent type *q* **do**20:                        Each agent $i\in N_{q}$ completes its local actor–critic update.21:                        Upload the required gradient and local-update information to the CC.22:                        The CC computes ${\bar{g}}_{q}^{t}$ and then evaluates $\delta_{i}^{t}$, $z_{i}^{t}$, and $I_{i}^{t}$ according to (48)–(50).23:                        The CC computes $p_{i}^{t}$ and selects $S_{q}^{t}$.24:                        Selected agents upload their complete local model updates.25:                        The CC computes $\omega_{i}^{t}$ and performs type-wise aggregation.26:                        Redistribute the updated type-specific global model.27:                  **end for**28:            **end if**29:      **end for**30:**end for**

#### 4.2.4. Theoretical Motivation

Let $F_{q}\left(\vartheta \right)$ be a differentiable type-specific learning objective with an $L_{q}$-Lipschitz continuous gradient. We define the aggregated direction as $d_{q}^{t}=\sum_{i\in S_{q}^{t}}\omega_{i}^{t}\left(\vartheta_{q,i}^{t+1}-\vartheta_{q}^{t}\right)$. The following standard descent argument clarifies the role of alignment and non-negative weighting.

**Proposition 1.**  *Suppose $\nabla F_{q}{\left(\vartheta_{q}^{t}\right)}^{⊤}d_{q}^{t}\leq -\kappa_{q}{∥d_{q}^{t}∥}_{2}^{2}$ for some $\kappa_{q}>0$. Then, an update ($\vartheta_{q}^{t+1}=\vartheta_{q}^{t}+\lambda d_{q}^{t}$) with $0<\lambda <2\kappa_{q}/L_{q}$ satisfies*

(53) $$F_{q}\left(\vartheta_{q}^{t+1}\right)\leq F_{q}\left(\vartheta_{q}^{t}\right)-\lambda \left(\kappa_{q}-\frac{L_{q}\lambda }{2}\right){∥d_{q}^{t}∥}_{2}^{2}.$$

**Proof.**  The $L_{q}$-smoothness inequality is applied to $\lambda d_{q}^{t}$, and the assumed alignment bound is substituted. □

The raw score favors agents whose historical gradients agree with the current group direction, while the scale penalty suppresses strongly fluctuating updates. The Softmax weights form a convex combination, so a selected update cannot acquire a negative coefficient. These properties reduce destructive interference and make the descent condition more plausible; they do not, by themselves, guarantee global convergence in the nonstationary multi-agent RL setting. A complete convergence analysis under function approximation and changing policies remains outside the scope of this work.

#### 4.2.5. Computational and Communication Complexity

For agent type *q*, let $P_{q}$ denote the number of trainable actor–critic parameters, $N_{q}$ denote the number of candidate agents, and $S_{q}$ denote the number of selected agents. Computing the contribution indicators requires $O(P_{q})$ operations per candidate, whereas type-wise aggregation requires $O(S_{q}P_{q})$ operations. Hence, the additional contribution-evaluation cost scales linearly with the model size and does not alter the dominant complexity of local actor–critic training.

The exact score in (49) requires the group mean gradient. In the centralized simulator, these gradients are already available to the training process. In a distributed deployment that explicitly uploads all gradients, the scoring traffic is $O(N_{q}P_{q})$, followed by $O(S_{q}P_{q})$ traffic for the selected model updates. Consequently, the exact implementation does not provide an asymptotic uplink-communication reduction over transmitting one update per participant. Secure aggregation or compressed gradient sketches could reduce this overhead, but they are not evaluated here. During inference, each agent executes only its local actor; therefore, federated scoring does not increase online inference complexity.

## 5. Performance Evaluation

In the previous section, we established the CHFAC design, including the action feasibility mappings, the online actor–critic training, and the significance-aware federated aggregation. We now turn from that design to an empirical validation of it. The remainder of this section first describes the simulation set-up and the compared benchmarks, then presents convergence, performance, ablation, sensitivity, and scalability results and finally reports the statistical validity of the documented gains.

This section evaluates the proposed CHFAC framework in a time-varying UAV-assisted vehicular sensing environment implemented using the Gym framework. The considered environment contains multiple vehicle sensing sources, UAV edge nodes, and a CC. VDs generate sensor-data packets according to a Poisson process, while UAVs move within the considered area to collect, process, and forward these packets. VDs move continuously during each episode according to the mobility model. Their positions, coverage relationships, and VD–UAV link conditions therefore change across time slots. The time variation in the environment arises from VD and UAV mobility, sensing-packet arrivals, queue evolution, concurrent transmissions, and agent decisions. Unless otherwise stated, all compared methods use the same VD mobility realizations, initial positions, environmental configurations, and episode lengths to ensure a fair comparison.

The default scenario contains six UAVs and thirty VDs in a 200m×200m virtual grid. The mean data volume of an active arrival is 3kb. Each UAV has a maximum movement distance of 6 m per slot, an observation radius of 60 m, a VD–UAV collection radius of 40 m, a VD–UAV maximum collection-service rate of 8kb/slot, and a processing rate of 20kb/slot. The UAV dropout probability is set to 0.005 per slot. The reward discount coefficient is 0.85, and the learning rates of the actor and critic networks are set to $10^{-3}$ and $2\times 10^{-3}$, respectively. Table 5 and Table 6 report the system and learning settings used by the implementation.

After training, each learned policy is evaluated over 1000 test episodes without exploration noise. The same environmental configurations and mobility scenarios are used for all compared methods. Unless otherwise stated, the reported results represent the mean and standard deviation across the 1000 test episodes. These statistics characterize the variability across the considered test scenarios.

The shaded regions in the training curves characterize a different source of variability. Specifically, the solid lines represent the pointwise mean obtained from independent repeated training runs, while the shaded regions represent the pointwise mean ± one standard deviation across these runs.

The performance of the proposed CHFAC is compared with three representative benchmarks to evaluate the gains from federated collaboration and significance-aware updating:

1. H-MAAC [33] is a federated multi-agent actor–critic method for age-sensitive MEC. Unlike CHFAC, it does not use the proposed significance-aware participant selection and contribution-weighted aggregation mechanism.
2. HF-MARL [34] is a heterogeneous federated multi-agent actor–critic method that performs periodic parameter sharing among independently trained agents of the same type. Since the original HF-MARL assumes stationary ground devices, it is adapted to the considered vehicular sensing environment using the same mobile-VD trajectories, system states, action constraints, communication conditions, and training horizon as CHFAC.
3. CHFAC without FL is an ablation that retains the same agent architectures, state and action spaces, reward function, and training settings as CHFAC while disabling federated updating. It quantifies the contribution of the FL component under otherwise identical conditions.

H-MAAC and HF-MARL are external benchmarks, whereas CHFAC without FL is an internal ablation. Together, they distinguish the gains from federated collaboration and the proposed significance-aware updating mechanism.

**Figure 4:**
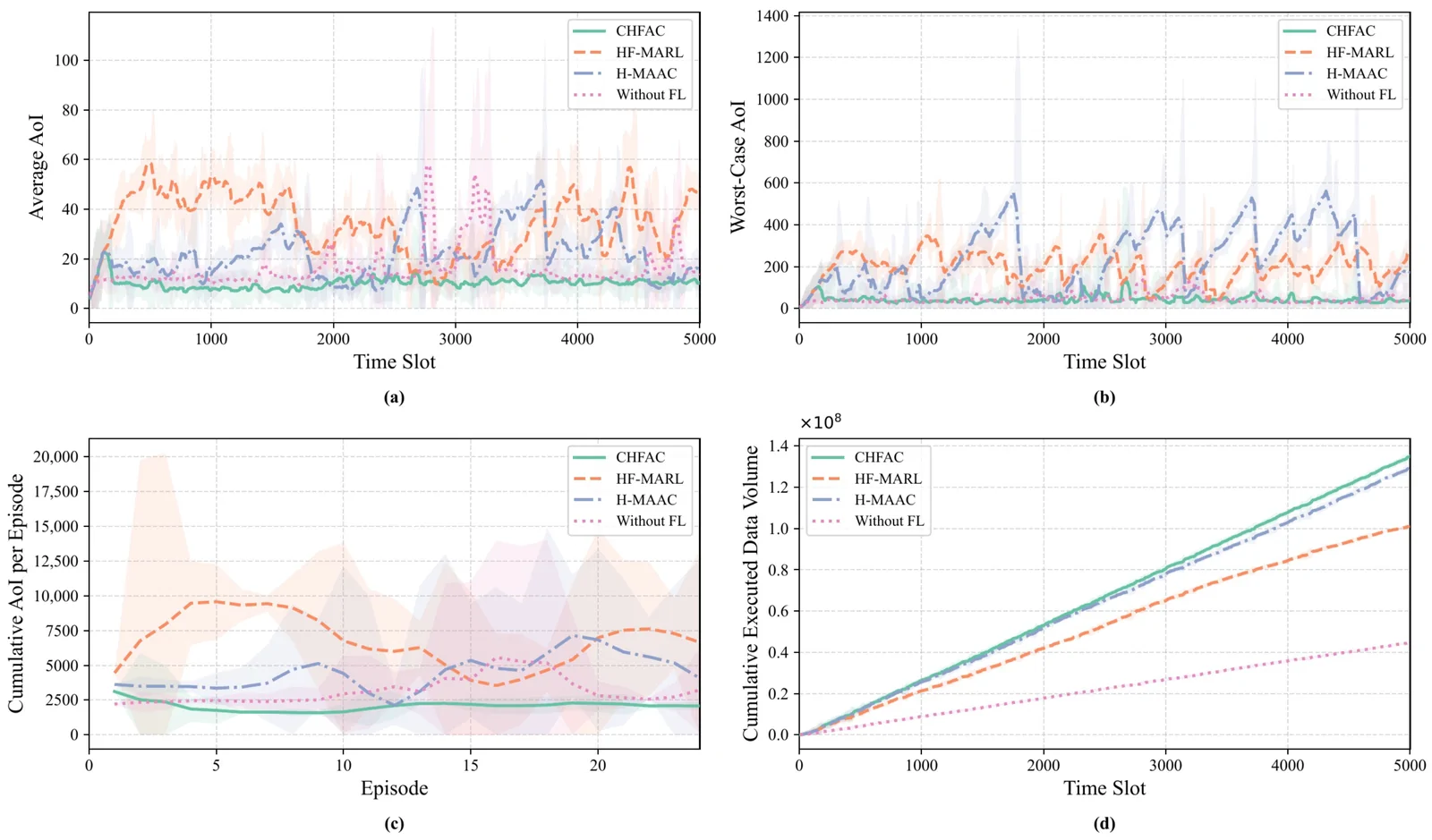
Performance evaluation of the proposed CHFAC algorithm compared with baseline methods. (**a**) Evolution of the system’s average AoI over time slots, comparing the proposed method against HF-MARL, H-MAAC, and Without FL baselines. (**b**) Evolution of the system’s worst-case AoI across different time slots for each algorithm. (**c**) Episode-level AoI curves during the training process, where a lower value indicates better information freshness. (**d**) Cumulative volume of executed data over time slots, illustrating the total amount of vehicular sensing data processed by the system.

Figure 4 displays the performance of the proposed CHFAC algorithm in comparison with three benchmark methods across multiple metrics. As shown in Figure 4a, the proposed algorithm generally maintains a lower and more stable system average AoI after the initial transient stage, highlighting its faster convergence and superior handling of network heterogeneity. Figure 4b shows the ability of our method to maintain a relatively low worst-case AoI and suppress most extreme AoI peaks when compared to the benchmarks. Figure 4c shows the episode-level AoI during training, where a lower value indicates better information freshness. CHFAC achieves a lower and more stable episode AoI than the benchmark methods. Lastly, Figure 4d illustrates the cumulative volume of executed data, where our approach demonstrates the largest completed-data volume, reflecting superior data-handling capabilities.

**Figure 5:**
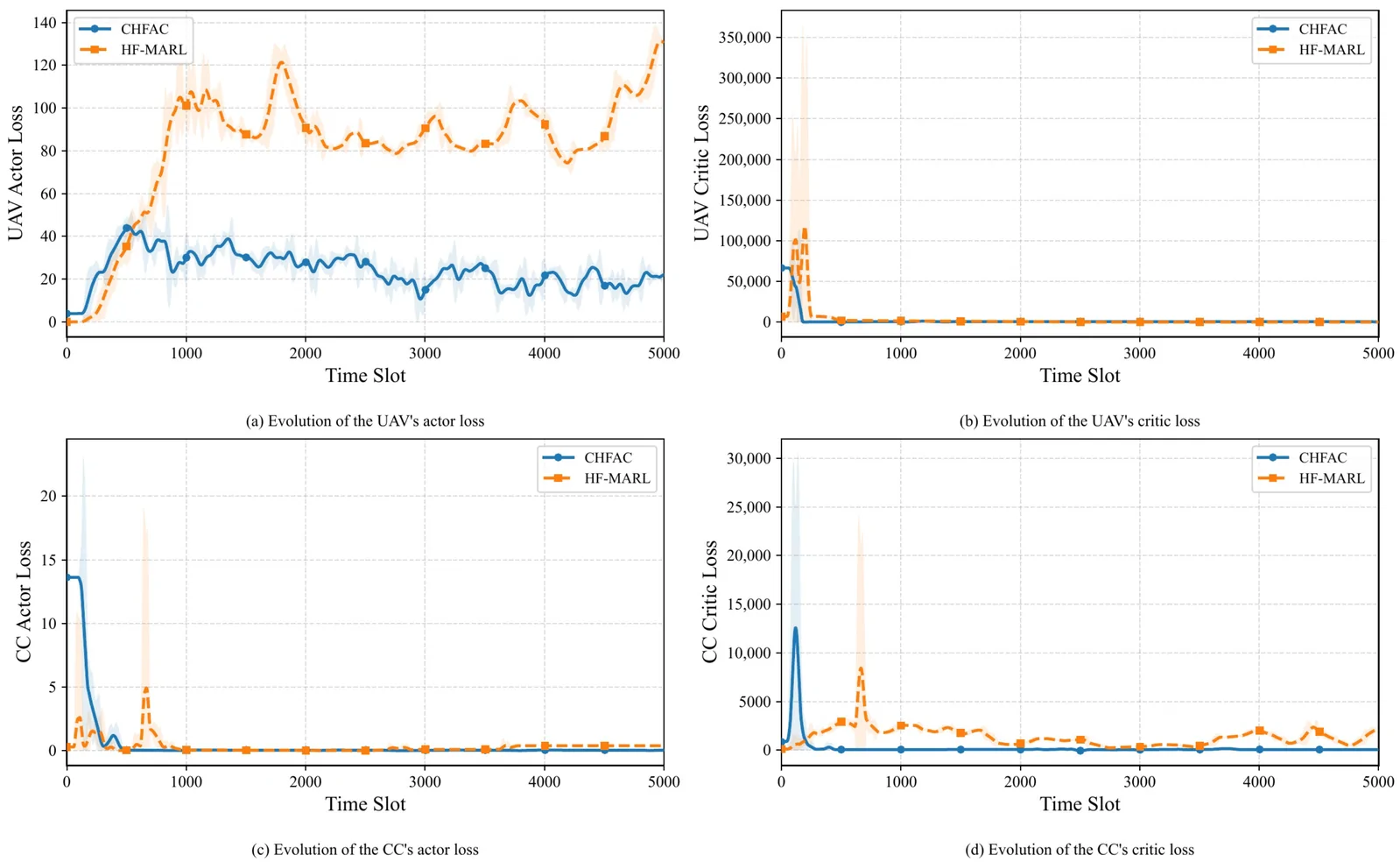
Training loss convergence curves of the actor–critic networks for different agents, comparing the proposed method with the HF-MARL baseline. (**a**) Evolution of the UAV agent’s actor network loss over time slots. (**b**) Evolution of the UAV agent’s critic network loss over time slots. (**c**) Evolution of the CC agent’s actor network loss during training. (**d**) Evolution of the CC agent’s critic network loss during training.

Figure 5 shows the training loss curves for the actor–critic networks, where our proposed method is compared against the HF-MARL baseline. At the beginning of the training, both methods exhibit fluctuating losses as they explore the environment. However, after the initial phase, a clear difference emerges. The loss values of our method exhibit different convergence patterns across the four network components. As shown in Figure 5a, the UAV actor loss of CHFAC remains clearly lower and more stable than that of HF-MARL after the initial training stage, whereas the HF-MARL loss remains high and continues to fluctuate. As shown in Figure 5b, the UAV critic losses of both methods undergo large initial fluctuations and subsequently decrease to values close to zero. Figure 5c shows that both CC actor losses converge toward zero, although CHFAC reaches a stable region earlier and maintains smaller residual fluctuations. As shown in Figure 5d, the CC critic loss of CHFAC rapidly decreases and remains close to zero, while HF-MARL retains larger fluctuations throughout the subsequent training process. This indicates that our method achieves more stable optimization, particularly for the UAV actor and CC critic networks, thereby supporting improved overall training stability.

**Figure 6:**
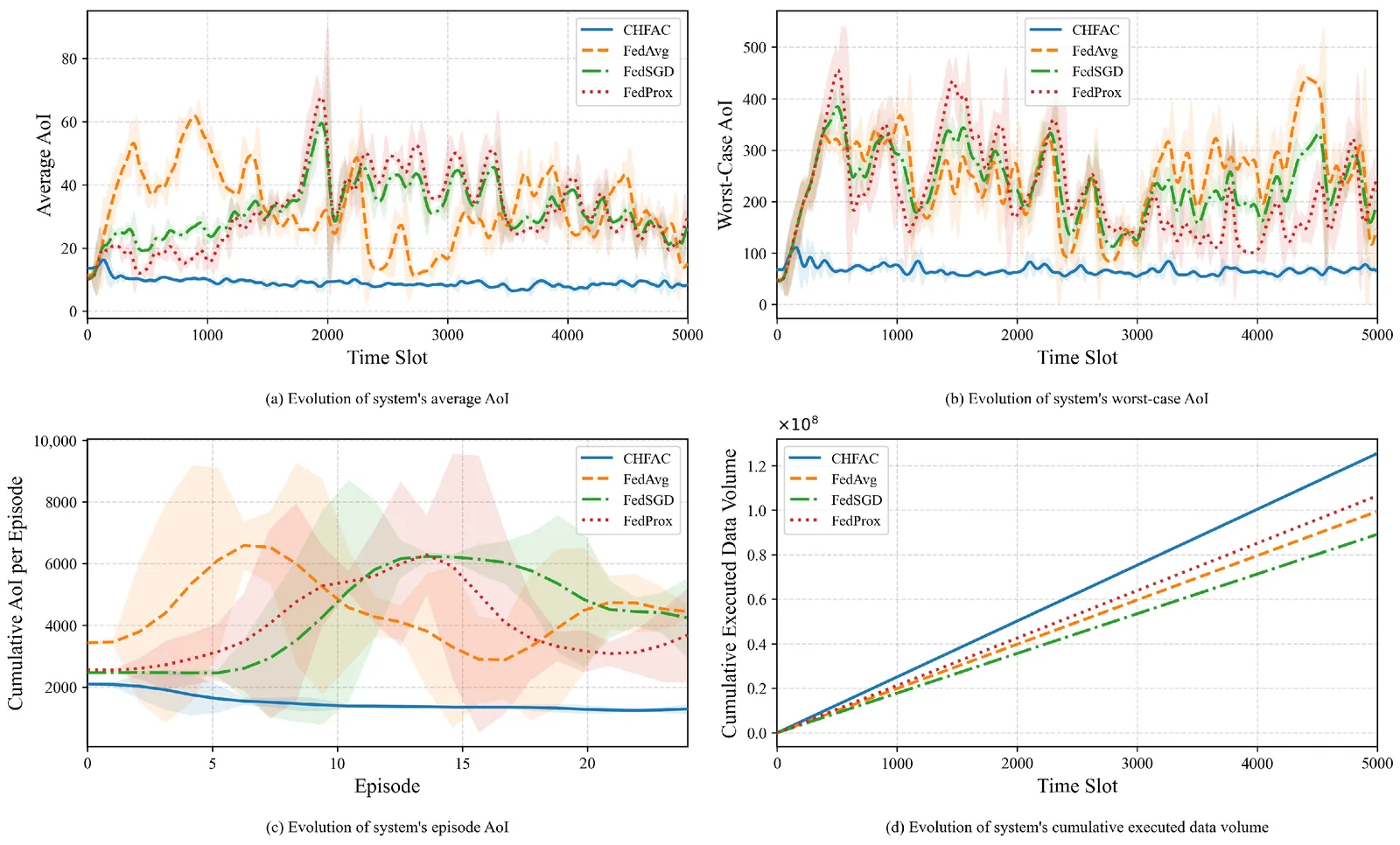
Performance comparison between the proposed FL algorithm and standard federated learning baselines (FedAvg, FedSGD, and FedProx). (**a**) Evolution of the system’s average AoI over time slots. (**b**) Evolution of the system’s worst-case AoI across time slots. (**c**) Evolution of the episode-level AoI during training, where a lower value indicates better information freshness. (**d**) Cumulative volume of executed data over time slots, illustrating the total amount of vehicular sensing data processed by the system.

Figure 6 compares CHFAC with the conventional FedAvg, FedSGD, and FedProx FL methods. Figure 6a,b show the evolution of the system’s average AoI and worst-case peak age. It can be seen that our method maintains consistently low AoI values. The baseline algorithms, however, result in higher and more variable AoI values, suggesting that they are less effective in maintaining information freshness. The episode-level AoI, as shown in Figure 6c, further supports this finding; our method obtains a lower and more stable episode-level AoI than the benchmark methods. Figure 6d further shows that CHFAC achieves the largest cumulative executed-data volume, indicating that it processes more vehicular sensing data within the same evaluation horizon.

**Figure 7:**
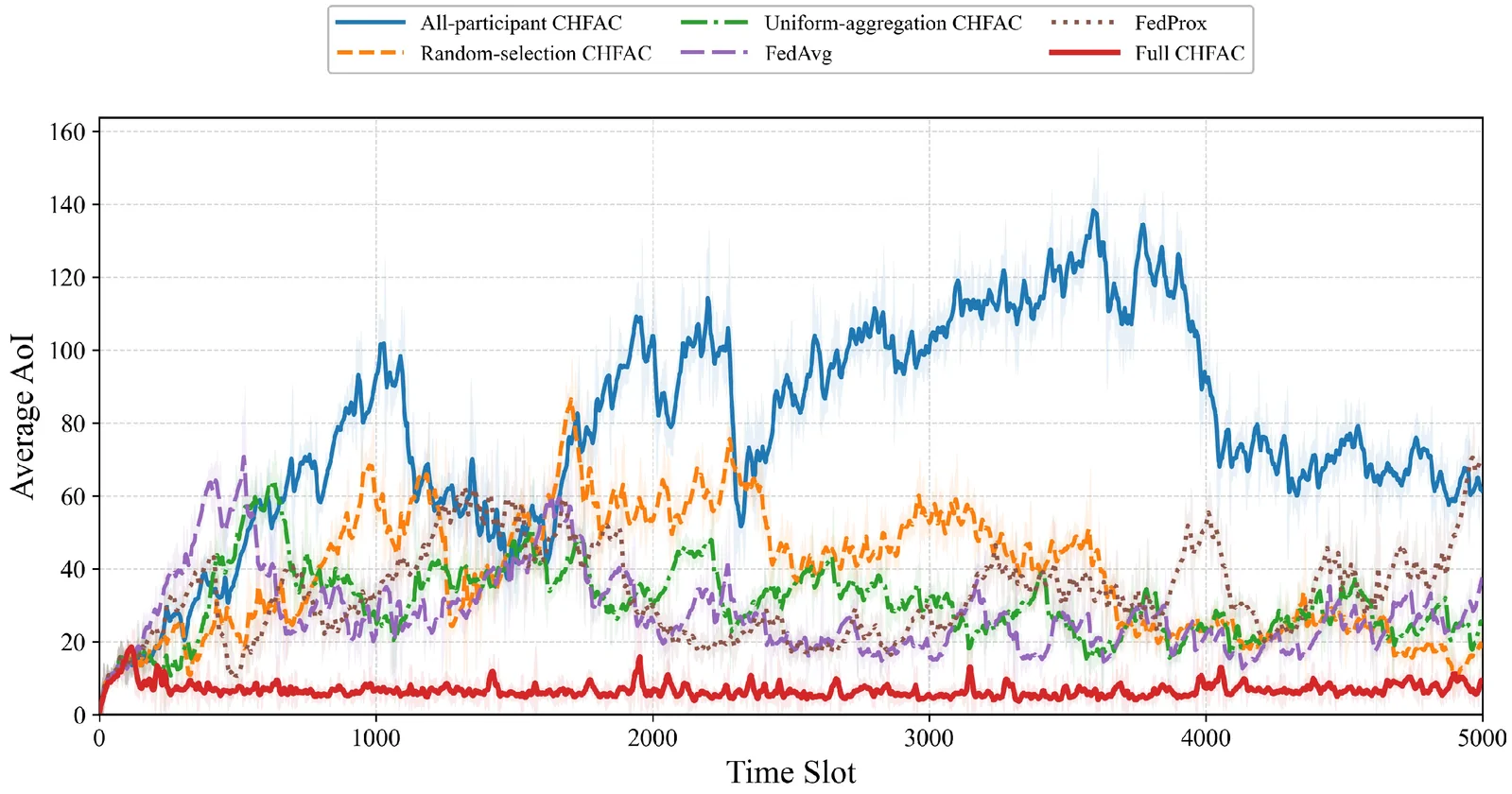
Ablation study of the participant-selection and model-aggregation mechanisms in CHFAC.

Figure 7 evaluates the effects of significance-aware participant selection and contribution-weighted aggregation. All variants use identical system and training settings. All-participant CHFAC includes all eligible agents, Random-selection CHFAC replaces significance-aware selection with random selection, and Uniform-aggregation CHFAC assigns equal weights to the selected updates. Full CHFAC maintains the lowest and most stable AoI throughout most of the training process. Random selection produces larger fluctuations, while uniform aggregation results in a consistently higher AoI than Full CHFAC. Moreover, including all participants causes pronounced instability, as low-quality or conflicting updates may adversely affect the global model. These results confirm that significance-aware selection and contribution-weighted aggregation jointly improve information freshness and training stability.

The ablation in Figure 7 isolates the two uses of the contribution score (participant selection and weighted aggregation). The score itself, however, combines three distinct components: the update-significance metric ($\delta_{i}^{t}$) in (48), the directional-alignment term (${\left({\bar{g}}_{q}^{t}\right)}^{⊤}g_{i}^{t-1}$), and the stability penalty ($\psi \;\delta_{i}^{t}{∥{\bar{g}}_{q}^{t}∥}_{2}^{2}$) in (49). To isolate the contribution of each pillar, we run three controlled experiments that isolate the effect of one component at a time while keeping the selection and aggregation protocol, the network architectures, the training budget, and the remaining experimental settings identical to the full CHFAC:

- Without update significance, the normalized metric ($\delta_{i}^{t}$) is replaced by the corresponding un-normalized effective-update magnitude, thereby removing the normalization-based significance measure while retaining agent-specific scale information in the stability term.
- Without directional alignment, the alignment term (${\left({\bar{g}}_{q}^{t}\right)}^{⊤}g_{i}^{t-1}$) is set to zero, so the score does not reward agreement with the current global learning direction.
- Without the stability penalty, the $\psi \;\delta_{i}^{t}{∥{\bar{g}}_{q}^{t}∥}_{2}^{2}$ term is removed, so updates are no longer penalized for large or volatile magnitudes.

Table 7 reports the resulting AoI metrics. Each pillar contributes to the overall gain: removing the directional-alignment term weakens the guidance of the global learning direction, removing the stability penalty admits unstable updates that destabilize the aggregated model, and removing the update-significance metric removes the scale awareness of the penalty. The full CHFAC achieves the lowest average and worst-case AoI.

**Figure 8:**
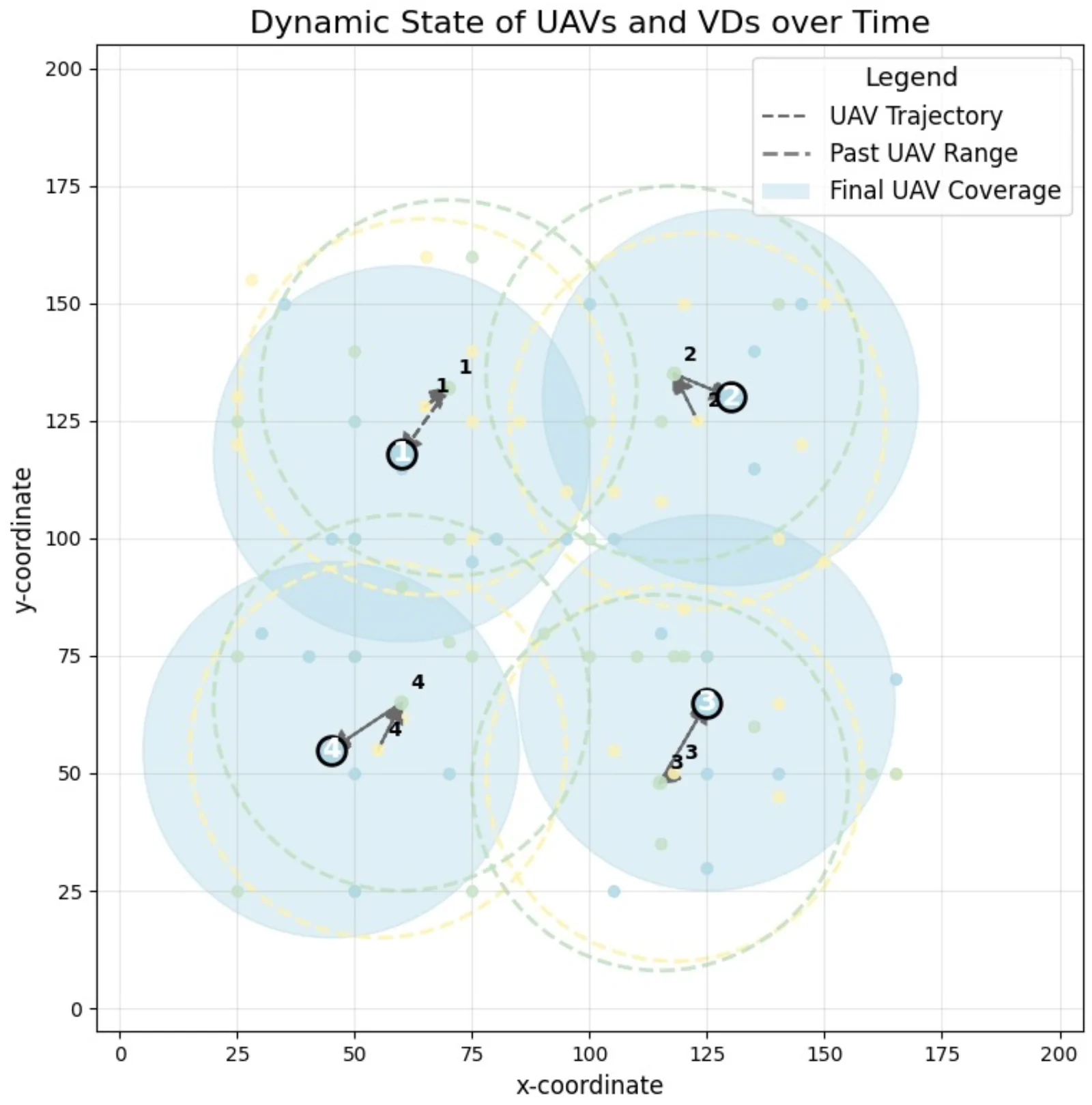
Learned UAV trajectories and cooperative coverage at three representative stages.

Figure 8 illustrates the UAVs’ trajectories under the proposed scheme, where the dashed lines represent past movement and the solid filled circles show the final coverage. Figure 8 shows that the UAVs dynamically adjust their positions to cover the changing spatial distribution of the VDs. The UAVs start from different initial positions and gradually form a stable cooperative formation that effectively serves the entire area. This visualization demonstrates that our algorithm successfully generates an intelligent and purposeful cooperative strategy in a dynamic environment.

**Figure 9:**
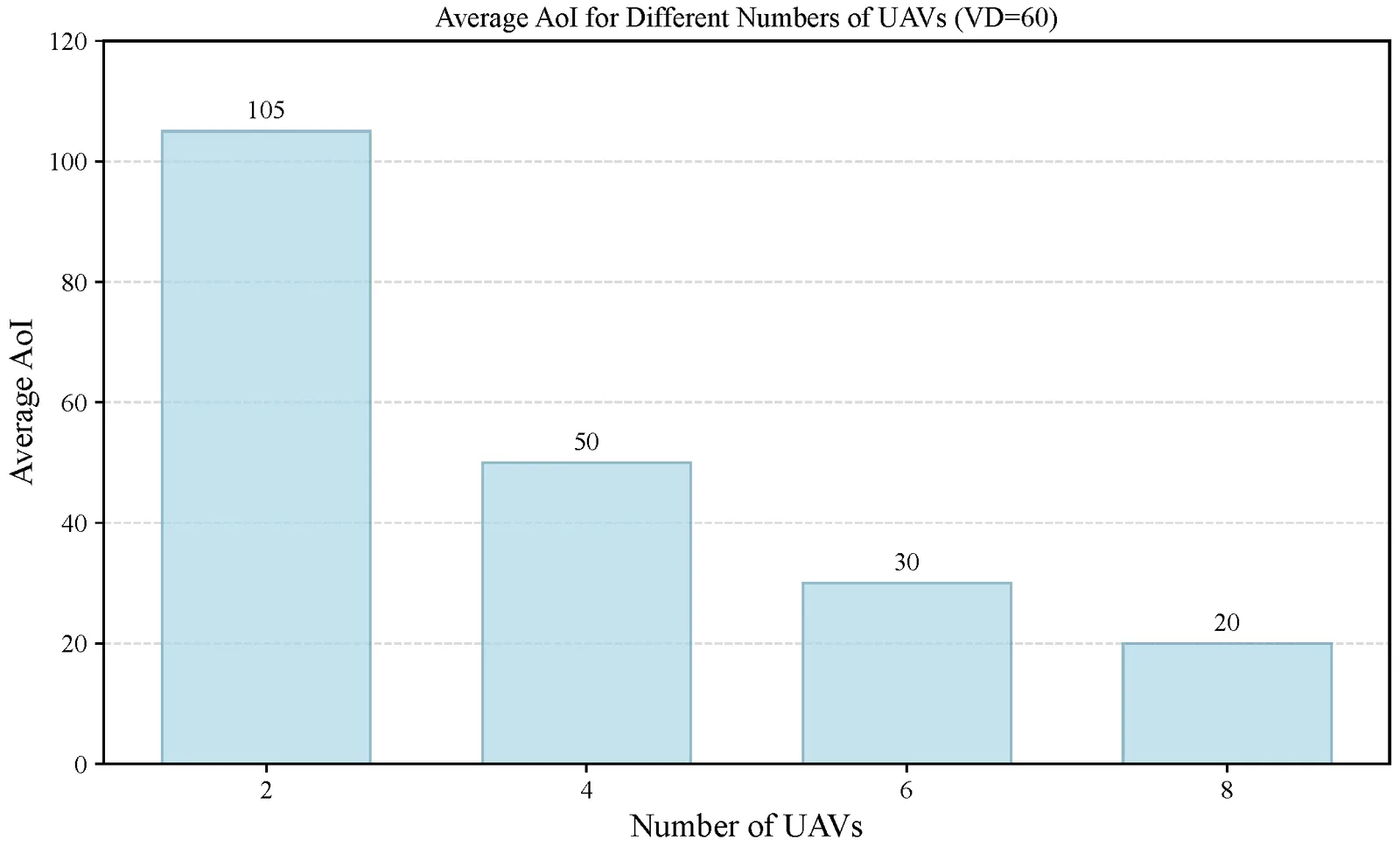
Impact of the number of UAVs on the average AoI (M=60).

**Figure 10:**
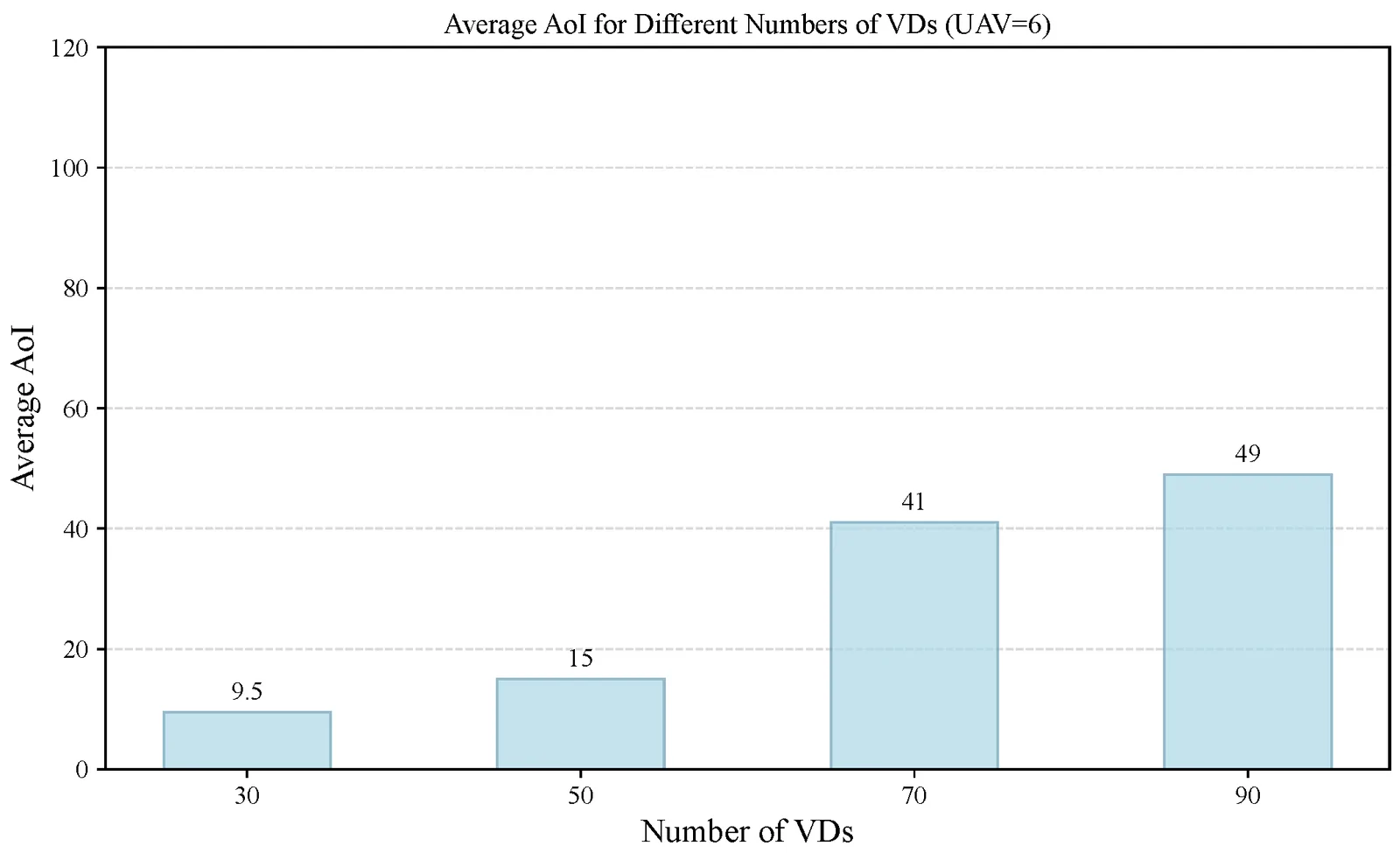
Impact of the number of VDs on the average AoI (K=6).

Figure 9 and Figure 10 show the impact of the number of UAVs and VDs on system performance, respectively. In Figure 9, the number of VDs is fixed at 60. As the number of UAVs increases, the average AoI of the system decreases markedly. This is because a larger number of UAVs can more effectively handle the communication and computation tasks, thereby improving the overall data freshness. Conversely, as shown in Figure 10, when the number of UAVs is fixed at six, increasing the system load by adding more VDs leads to a gradual rise in the average AoI. These results align with theoretical expectations and demonstrate the system’s scalability and its trade-offs.

To examine the robustness of CHFAC to its key federation coefficients, we conduct a controlled sensitivity analysis of the proximal coefficient (μ) and the stability-penalty coefficient (ψ). Each coefficient is varied over {0,0.01,0.05,0.1,0.2}, while the other coefficient is fixed at its default value of 0.1. All remaining system parameters, training budgets, and evaluation settings are kept unchanged. Each trained configuration is evaluated over 1000 exploration-free test episodes. The markers and error bars report the mean and standard deviation across these test episodes, respectively.

**Figure 11:**
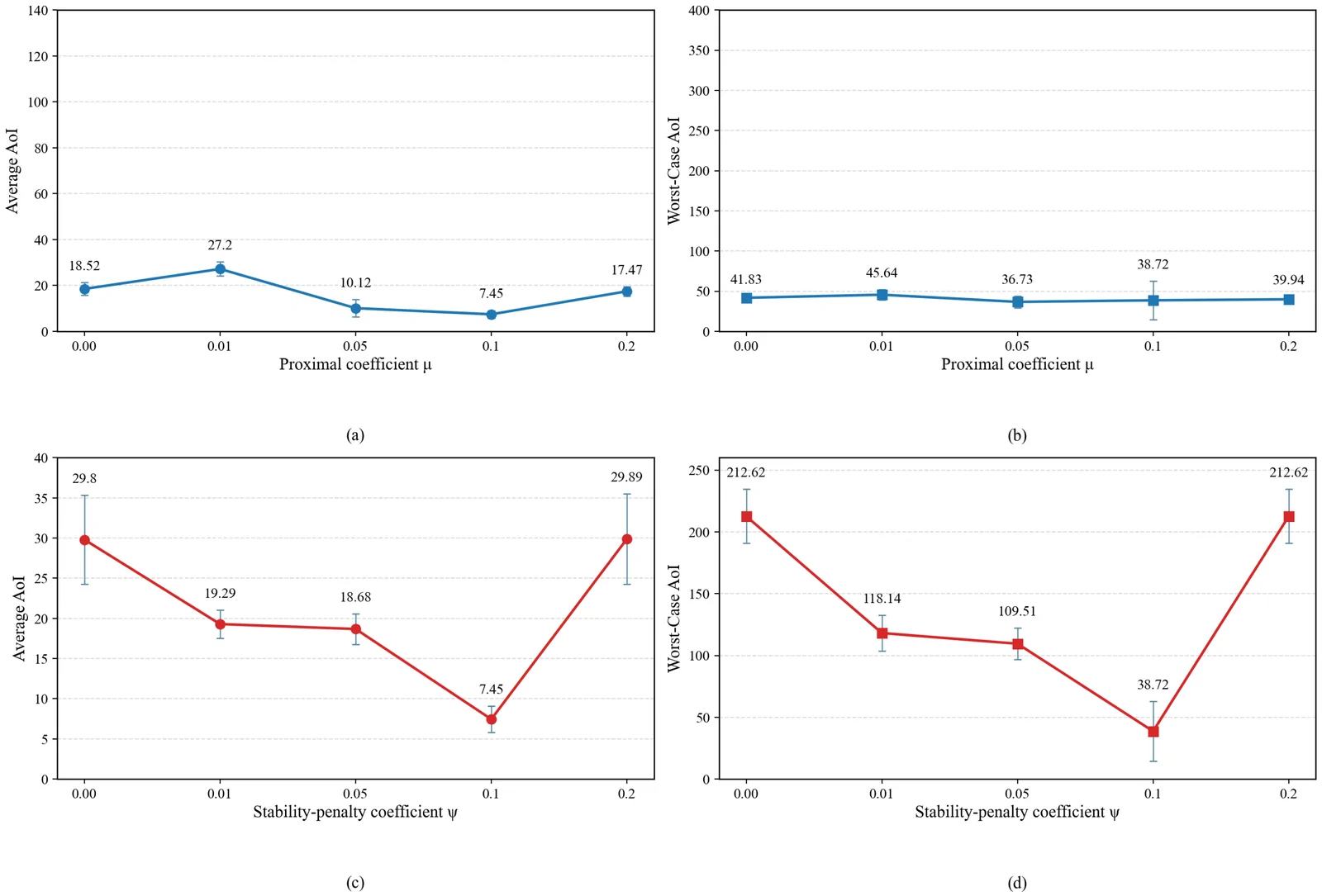
Sensitivity analysis of the key federation coefficients. (**a**) Average AoI under different proximal coefficients (μ). (**b**) Worst-case AoI under different proximal coefficients (μ). (**c**) Average AoI under different stability-penalty coefficients (ψ). (**d**) Worst-case AoI under different stability-penalty coefficients (ψ).

As shown in Figure 11a,b, the influence of μ is non-monotonic. Setting μ=0.1 yields the lowest average AoI of 7.45 and the lowest worst-case AoI of 38.72 among the examined values. A small or zero proximal coefficient does not consistently constrain local model drift, whereas an excessively large coefficient restricts the adaptation of local policies to heterogeneous observations and system states. Therefore, μ=0.1 provides an effective balance between local adaptation and global-model consistency.

Figure 11c,d show a clearer sensitivity to ψ. Without the stability penalty, i.e., ψ=0, the average and worst-case AoI increase to 29.80 and 212.62, respectively. Increasing ψ to 0.1 reduces these values to 7.45 and 38.72. However, further increasing ψ to 0.2 degrades the performance because an excessive penalty may suppress informative model updates with relatively large magnitudes. These results indicate that ψ=0.1 effectively filters unstable updates without excessively restricting useful local contributions. The sensitivity results therefore support the default settings of μ=0.1 and ψ=0.1 reported in Table 6.

Finally, Table 8 presents the numerical AoI results over 1000 test episodes. The standard deviations reported in Table 8 are calculated across the 1000 exploration-free post-training test episodes, characterizing the variability among the evaluated test scenarios. CHFAC achieves an average AoI of 7.45±1.65 and a worst-case AoI of 38.72±24.06. In comparison, HF-MARL, H-MAAC, and CHFAC without FL achieve average AoI values of 35.45±10.26, 20.64±9.56, and 10.52±1.49, respectively. Therefore, CHFAC reduces the average AoI by 79.0%, 63.9%, and 29.2% relative to the three methods. These results demonstrate that federated collaboration and significance-aware updating improve the freshness and stability of vehicular sensing information.

To further substantiate the performance improvements reported in Table 8, we conduct paired statistical tests using the same 1000 exploration-free post-training test episodes. For each matched test episode, all compared methods are evaluated under the same environmental configuration, VD mobility realization, packet-arrival realization, initial system state, and evaluation horizon. Therefore, the episode-level AoI values are obtained by CHFAC and each benchmark form paired observations.

We apply a two-sided Wilcoxon signed-rank test to each pairwise comparison between CHFAC and the corresponding benchmark. The null hypothesis is that the median of the paired AoI differences is zero. As a complementary parametric analysis, a two-sided paired *t*-test is also conducted on the same matched episode-level observations. A *p*-value below 0.05 is considered statistically significant at the 5% significance level.

The results are summarized in Table 9. The paired statistical tests show that the average AoI achieved by CHFAC is significantly different from that of HF-MARL, H-MAAC, and Without FL, providing statistical support for the performance improvements reported in Table 8.

## 6. Conclusions

This paper investigated AoI optimization of vehicular sensing in a three-layer UAV-assisted edge network comprising VDs, multiple UAVs, and a CC. VDs periodically generate sensor-data packets, UAVs provide mobile edge processing and data relay services, and the CC coordinates system-wide resource allocation. To jointly optimize sensor-data transmission, UAV movement, edge packet processing, computation offloading, and bandwidth allocation, we propose a CHFAC framework. Its significance-aware FL mechanism evaluates local model updates according to update significance, alignment with the global learning direction, and training stability and uses the resulting contribution scores for non-uniform agent selection and contribution-weighted aggregation. Simulation results show that CHFAC achieves the lowest average AoI, substantially reduces the worst-case AoI relative to HF-MARL and H-MAAC, and achieves worst-case performance comparable to the non-federated baseline. Over 1000 test episodes, CHFAC achieved an average AoI of 7.45±1.65 and a worst-case AoI of 38.72±24.06, reducing the average AoI by 79.0%, 63.9%, and 29.2% compared with HF-MARL, H-MAAC, and the non-federated ablation, respectively. The learned UAV trajectories further demonstrate that UAV mobility can be adaptively coordinated with the spatial distribution of vehicular sensing sources, confirming the effectiveness of CHFAC in maintaining timely vehicular sensing information in dynamic UAV-assisted edge networks. Although the proposed CHFAC framework achieves effective AoI optimization, several limitations remain. The current model focuses on network-level UAV mobility and does not consider detailed flight dynamics or propulsion energy constraints. In addition, the evaluation is based on simulation environments, and future work will investigate more realistic mobility traces, imperfect observations, and large-scale deployments. 

## Figures and Tables

**Table 1:** Comparison of existing studies in the Related Work.

References	UAV-Assisted Edge	AoI/Freshness	Joint Optimization	RL/MARL	FL	Significance-Aware Updating
[6]	🗸	×	🗸	🗸	×	×
[7]	🗸	×	🗸	×	×	×
[9]	×	🗸	×	×	×	×
[10]	×	🗸	🗸	🗸	×	×
[11]	×	×	×	🗸	×	×
[13]	×	×	×	×	🗸	×
[14]	×	×	🗸	×	×	×
[15]	🗸	×	🗸	×	×	×
[16]	🗸	×	🗸	×	×	×
[17]	🗸	×	🗸	×	×	×
[18]	🗸	×	🗸	×	×	×
[22]	×	🗸	×	×	×	×
[23]	🗸	🗸	🗸	🗸	×	×
[24]	🗸	🗸	×	×	×	×
[25]	🗸	🗸	🗸	×	×	×
[26]	🗸	🗸	🗸	×	×	×
[27]	×	×	🗸	🗸	×	×
[28]	×	×	🗸	🗸	×	×
[29]	×	×	×	🗸	×	×
[32]	×	×	×	×	🗸	×
[33]	×	🗸	🗸	🗸	🗸	×
[34]	🗸	🗸	🗸	🗸	🗸	×
[35]	×	×	🗸	🗸	🗸	×
[36]	×	×	×	×	🗸	×
Proposed	🗸	🗸	🗸	🗸	🗸	🗸

**Table 2:** Main notations.

Symbol	Description
*t*	The *t*-th time slot.
*K*	Number of UAVs.
*M*	Number of VDs.
G=M+K+1	Number of heterogeneous agents.
$d_{m}\left(t\right)$	Fraction of VD *m*’s packet assigned to local computation.
$L_{m}\left(t\right)$	Size of the sensor-data packet generated by VD *m*.
${\mathrm{exec}}_{k}\left(t\right)$	Task execution decision vector for UAV *k*.
${\mathrm{off}}_{k}\left(t\right)$	Offloading decision vector for UAV *k*.
Δ(t)	AoI of the system at time *t*.
$A_{g},A_{g}^{\prime },C_{g},C_{g}^{\prime }$	Actor/critic networks for learning agents.
$A=\{A_{1},\cdots ,A_{G}\}$	Set of actor networks.
$C=\{C_{1},\cdots ,C_{G}\}$	Set of critic networks.
$\theta_{g},\theta_{g}^{\prime },\phi_{g},\phi_{g}^{\prime }$	Neural network parameters.
$\eta_{A},\eta_{C}$	Learning rates for actor/critic networks.
B	Experience replay buffer.
γ∈[0,1]	Reward discount factor.
τ∈[0,1]	Target network update coefficient.

**Table 3:** Parameterized actor–critic network architectures of the heterogeneous agents in CHFAC.

Agent	Network	Input Shape	Feature Processing	Output Head
VD	Actor	$O_{m}\left(t\right)\in R^{2\times B_{m,\max}+2}$	Transpose–Flatten; Dense (1,ReLU)	Dense (1,Sigmoid) for $d_{m}\left(t\right)$
	Critic	$\left[O_{m}\left(t\right),d_{m}\left(t\right)\right]$; $2B_{m,\max}+3$ scalar inputs	Observation Flatten; Dense (1,ReLU); observation–action concatenation	Dense (1,Linear) for $Q_{m}$
UAV	Actor	Spatial map: $N_{x}^{s}\times N_{y}^{s}\times C_{s}$; collection buffer: $2\times B_{k,\max}^{\mathrm{col}}$; executed buffer: $2\times B_{k,\max}^{\mathrm{exe}}$; bandwidth feature: 1	Conv2D (2,3×3,ReLU); AvgPool (9×9); AlphaDropout (0.2); buffer-specific Dense branches; spatial–buffer feature fusion	Movement-score map: $N_{x}^{a}\times N_{y}^{a}$; execution: Dense ($B_{k,\max}^{\mathrm{col}}+1$, Softmax); offloading: Dense ($B_{k,\max}^{\mathrm{exe}}+1$, Softmax)
	Critic	UAV actor observation; movement map: $N_{x}^{a}\times N_{y}^{a}$; execution: $B_{k,\max}^{\mathrm{col}}+1$; offloading: $B_{k,\max}^{\mathrm{exe}}+1$	Dense (1)–Conv2D (1,3×3); AvgPool (6×6)–Flatten–Dense (2); buffer/action feature extraction; feature concatenation	Dense (1,Linear) for $Q_{k}$
CC	Actor	UAV buffer features: $K\times (B_{k,\max}^{\mathrm{col}}+B_{k,\max}^{\mathrm{exe}})$; UAV positions: K×2	Buffer Dense (1,ReLU); position Dense (2,ReLU); feature concatenation; fusion Dense (1,ReLU)	Dense (K,Softmax) for b(t)
	Critic	CC actor observation; bandwidth vector: *K*	Buffer Dense (1)–Dense (1); position Dense (1); observation–action concatenation	Dense (1,Linear) for $Q_{c}$

**Table 4:** Notation for the significance-aware FL mechanism.

Symbol	Description
*t*	Index for the global communication round.
$N_{q}$	Set of candidate agents in federation group q∈{VD,UAV}.
$N_{q}$	Number of candidate agents of type *q*.
$S_{q}$	Number of selected agents of type *q*.
$S_{q}^{t}$	Set of selected agents of type *q* at federated round *t*.
$\vartheta_{q}^{t}$	Global model parameters of federation group *q* at the start of round *t*.
$\vartheta_{q,i}^{t+1}$	Local model parameters of agent *i* after training.
$F_{i}\left(\cdot \right)$	Local loss function for agent *i*.
$H_{i}\left(\cdot \right)$	Regularized local objective function for agent *i*.
$g_{i}^{t}$	Local gradient $\nabla F_{i}\left(\vartheta_{q}^{t}\right)$ at the start of round *t*.
$g_{i}^{t-1}$	Local gradient from the previous round, t−1.
${\bar{g}}_{q}^{t}$	Average gradient of federation group *q* at round *t*.
$\delta_{i}^{t}$	Normalized effective-update scale of agent *i*.
$z_{i}^{t}$	Raw alignment–stability score of agent *i*.
$I_{i}^{t}$	Non-negative contribution value of agent *i*.
$p_{i}^{t}$	Selection probability for agent *i*.
$\omega_{i}^{t}$	Aggregation weight of selected agent *i*.
μ,ψ,β	Proximal, stability-penalty, and Softmax coefficients.

**Table 5:** System and communication parameters.

Parameter	Symbol	Value
Simulation area	Ω	200m×200m
Number of UAVs	*K*	6
Number of VDs	*M*	30
Sensor-data arrival process	–	Poisson process
Mean generated data per active arrival	λ	3 kb
Packet-size rejection threshold	$L_{\max}$	6 kb
Slot duration	$T_{s}$	1 s
Maximum UAV movement distance per slot	$r_{\mathrm{move}}^{k}$	6 m
UAV observation radius	$R_{\mathrm{obs}}$	60 m
VD–UAV collection radius	$R_{\mathrm{col}}$	40 m
VD–UAV maximum collection-service rate	$r_{\mathrm{VD}}^{\max}$	8 kb/slot
UAV processing rate	$f_{k}^{c}$	20 kb/slot
VD source-buffer capacity	$B_{m,\max}$	20 packets
UAV buffer capacity	$B_{k,\max}^{\mathrm{col}}/B_{k,\max}^{\mathrm{exe}}$	10 packets
UAV altitude	*H*	100 m
Carrier frequency	$f_{c}$	2.4 GHz
UAV transmit power	$P_{k}^{\mathrm{tr}}$	0.2 W
UAV–CC bandwidth	*W*	8 MHz
Noise power spectral density	$N_{0}$	$10^{-13}$ W/Hz
LoS probability parameters	a,b	9.61,0.16
Additional LoS/NLoS losses	$\eta^{L},\eta^{\mathrm{NL}}$	1,20 dB

**Table 6:** Learning and federation parameters.

Parameter	Symbol	Value
Training interaction slots	$T_{\mathrm{train}}$	5000
Logging/evaluation window	–	200 slots
Number of test episodes	–	1000
Optimizer	–	Adam
Actor learning rate	$\eta_{A}$	$10^{-3}$
Critic learning rate	$\eta_{C}$	$2\times 10^{-3}$
Discount factor	γ	0.85
Initial/minimum exploration probability	$\epsilon_{0}/\epsilon_{\min}$	0.2/0.05
Exploration decay every 200 slots	–	0.9
Mini-batch size	–	64
Retained replay window per agent	–	128 transitions
Target-network update interval	$T_{\mathrm{up}}$	8 slots
Target-update/retention coefficient	τ/(1−τ)	0.2/0.8
Federation interval	$E_{f}$	8 slots
Federation start slot	–	100
Participant ratio	ρ	0.5
Selected VDs/UAVs per round	$S_{\mathrm{VD}}/S_{\mathrm{UAV}}$	15/3
Proximal coefficient	μ	0.1 (0 for FedAvg)
Stability-penalty coefficient	ψ	0.1
Softmax coefficient	β	1.0

**Table 7:** Component-level ablation of the significance-aware mechanism.

Variant	AoI	
	**Average**	**Worst Case**
Full CHFAC	7.45 ± 1.65	38.72 ± 24.06
Without update significance ($\delta_{i}^{t}$)	18.52 ± 2.83	41.83 ± 2.09
Without directional alignment	29.91 ± 6.37	199.16 ± 64.96
Without stability penalty	29.80 ± 5.54	212.62 ± 21.87

**Table 8:** Numerical comparison of AoI metrics.

Approach	AoI	Worst-Case AoI
HF-MARL	35.45±10.26	206.46±59.60
H-MAAC	20.64±9.56	280.57±178.46
Without FL	10.52±1.49	37.42±16.61
CHFAC	7.45±1.65	38.72±24.06

**Table 9:** Paired statistical comparison of the average AoI over 1000 matched post-training test episodes.

Method	Mean Diff. (CHFAC − Method)	Wilcoxon *p*	Paired *t p*
HF-MARL	−28.00	$1.91\times 10^{-6}$	$1.12\times 10^{-10}$
H-MAAC	−13.19	$1.91\times 10^{-6}$	$7.59\times 10^{-6}$
Without FL	−3.07	$1.91\times 10^{-6}$	$7.84\times 10^{-10}$

## Data Availability

All data generated or analyzed during this study are included in this published article. Further information is available from the corresponding author upon reasonable request.

## Abbreviations

The following abbreviations are used in this manuscript:

MEC	Mobile edge computing
VEC	Vehicular edge computing
IoV	Internet of Vehicles
IoT	Internet of Things
UAV	Unmanned aerial vehicle
AoI	Age of information
DRL	Deep reinforcement learning
MARL	Multi-agent reinforcement learning
FL	Federated learning
V2X	Vehicle to everything
CHFAC	Collaborative heterogeneous federated actor–critic
FRL	Federated reinforcement learning
CC	Cloud center
A2G	Air to ground
LoS	Line of sight
NLoS	Non-line of sight
SINR	Signal-to-interference-plus-noise ratio
VD	Vehicle device
DEC-POMDP	Decentralized partially observable Markov decision process
RL	Reinforcement learning
DDPG	Deep deterministic policy gradient
A2C	Advantage actor–critic
MLP	Multilayer perceptron
CNN	Convolutional neural network
SGD	Stochastic gradient descent
H-MAAC	Heterogeneous multi-agent actor–critic
HF-MARL	Heterogeneous federated multi-agent reinforcement learning
